# Aqua­dipicrato(tetra­ethyl­ene glycol)gadolinium(III) picrate methanol hemisolvate

**DOI:** 10.1107/S1600536808026147

**Published:** 2008-08-16

**Authors:** Eny Kusrini, Muhammad Idiris Saleh, Reza Kia, Hoong-Kun Fun

**Affiliations:** a School of Chemical Sciences, Universiti Sains Malaysia, 11800 USM, Penang, Malaysia; b X-ray Crystallography Unit, School of Physics, Universiti Sains Malaysia, 11800 USM, Penang, Malaysia

## Abstract

The asymmetric unit of the title compound [systematic name: aqua­bis(2,4,6-trinitro­phenolato)(3,6,9-trioxaundecane-1,11-diol)gadolinium(III) 2,4,6-trinitro­phenolate methanol hemi­solvate], [Gd(C_6_H_2_N_3_O_7_)_2_(C_8_H_18_O_5_)(H_2_O)](C_6_H_2_N_3_O_7_)·0.5CH_4_O,  contains two crystallographically independent Gd^III^ complex cations with two uncoordinated picrate anions and one methanol mol­ecule. Each Gd^III^ atom has nine coordination sites occupied by five O atoms of tetra­ethyl­ene glycol as a penta­dentate ligand, one O atom of a water mol­ecule and three O atoms of the two picrate anions as bidentate and monodentate ligands. The geometry is distorted tricapped trigonal prismatic. The mean planes of the two coordinated mono- and bidentate picrate ligands to the Gd^III^ center are almost perpendicular to each other, as indicated by the dihedral angles of 89.92 (8) and 86.60 (8)° in the two complex cations. The ions are arranged in a two-dimensional network parallel to the *ac* plane. Short O⋯O and N⋯O contacts between the nitro groups, intra­molecular C—H⋯O hydrogen bonds, inter­molecular O—H⋯O, O—H⋯N and C—H⋯O hydrogen bonds, and two π–π inter­actions between benzene rings [centroid–centroid distances = 3.8073 (10)–3.9831 (10) Å] are observed. The methanol solvent mol­ecule is disordered over two positions, with site-occupancy factors of *ca* 0.6 and 0.4.

## Related literature

For hydrogen-bond motifs, see: Bernstein *et al.* (1995 ▶). For bond-length data, see: Allen *et al.* (1987 ▶, 1998 ▶); For related literature, see, for example: Kusrini *et al.* (2008 ▶); Rogers, Rollins *et al.* (1991 ▶); Rogers, Russel *et al.* (1991 ▶); Rogers & Henry (1992 ▶); Rogers *et al.* (1997 ▶); Casellato *et al.* (1982 ▶).
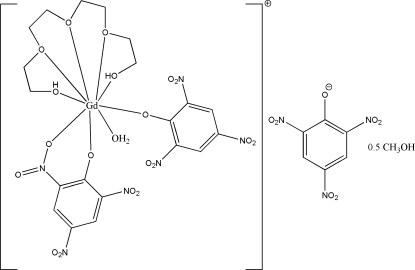


## Experimental

### 

#### Crystal data

[Gd(C_6_H_2_N_3_O_7_)_2_(C_8_H_18_O_5_)(H_2_O)](C_6_H_2_N_3_O_7_)·0.5CH_4_O
*M* *_r_* = 1069.83
Triclinic, 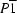

*a* = 8.1816 (6) Å
*b* = 18.6657 (14) Å
*c* = 24.9646 (18) Å
α = 104.188 (3)°
β = 96.445 (4)°
γ = 95.483 (4)°
*V* = 3642.7 (5) Å^3^
*Z* = 4
Mo *K*α radiation
μ = 1.94 mm^−1^
*T* = 100.0 (1) K
0.51 × 0.31 × 0.16 mm

#### Data collection

Bruker SMART APEXII CCD area-detector diffractometer
Absorption correction: multi-scan (**SADABS**; Bruker, 2005 ▶) *T*_min_ = 0.432, *T*_max_ = 0.736
125488 measured reflections
38087 independent reflections
30033 reflections with *I* > 2σ(*I*)
*R*_int_ = 0.041

#### Refinement

*R*[*F* ^2^ > 2σ(*F* ^2^)] = 0.033
*wR*(*F* ^2^) = 0.092
*S* = 1.08
38087 reflections
1158 parameters
2 restraints
H-atom parameters constrained
Δρ_max_ = 1.66 e Å^−3^
Δρ_min_ = −1.65 e Å^−3^

### 

Data collection: *APEX2* (Bruker, 2005 ▶); cell refinement: *APEX2*; data reduction: *SAINT* (Bruker, 2005 ▶); program(s) used to solve structure: *SHELXTL* (Sheldrick, 2008 ▶); program(s) used to refine structure: *SHELXTL*; molecular graphics: *SHELXTL*; software used to prepare material for publication: *SHELXTL* and *PLATON* (Spek, 2003 ▶).

## Supplementary Material

Crystal structure: contains datablocks global, I. DOI: 10.1107/S1600536808026147/is2323sup1.cif

Structure factors: contains datablocks I. DOI: 10.1107/S1600536808026147/is2323Isup2.hkl

Additional supplementary materials:  crystallographic information; 3D view; checkCIF report

## Figures and Tables

**Table 1:** Selected interatomic distances (Å)

N2*A*⋯O22*B*^i^	2.948 (3)
N2*B*⋯O25*A*^ii^	2.916 (2)
O9*B*⋯O25*A*^ii^	2.924 (2)
O17*B*⋯O24*B*^iii^	3.035 (3)

Symmetry codes: (i) 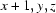; (ii) 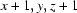; (iii) 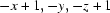.

**Table 2:** Hydrogen-bond geometry (Å, °)

*D*—H⋯*A*	*D*—H	H⋯*A*	*D*⋯*A*	*D*—H⋯*A*
O1*A*—H1*OA*⋯O9*A*^iv^	0.93	2.18	2.822 (2)	125
O1*A*—H1*OA*⋯O10*A*^iv^	0.93	2.23	3.156 (2)	173
O1*A*—H1*OA*⋯N2*A*^iv^	0.93	2.51	3.374 (2)	154
O5*A*—H5*OA*⋯O20*A*^i^	0.85	1.92	2.706 (2)	153
O5*A*—H5*OA*⋯O21*A*^i^	0.85	2.58	3.132 (2)	123
O1*WA*—H1*WA*⋯O26*A*^i^	0.85	2.54	2.879 (2)	105
O1*WA*—H1*WA*⋯N6*A*	0.85	2.56	3.344 (2)	154
O1*WA*—H2*WA*⋯O20*A*^i^	0.85	1.84	2.686 (2)	173
O1*B*—H1*OB*⋯O20*B*^i^	0.84	1.88	2.661 (2)	155
O5*B*—H5*OB*⋯O9*B*^v^	0.87	1.95	2.805 (2)	168
O5*B*—H5*OB*⋯N2*B*^v^	0.87	2.62	3.451 (2)	160
O1*WB*—H1*WB*⋯O21*B*^i^	0.87	2.39	2.727 (2)	104
O1*WB*—H2*WB*⋯O20*B*^i^	0.86	1.74	2.592 (2)	170
O1*WB*—H2*WB*⋯O21*B*^i^	0.86	2.42	2.727 (2)	101
O1*X*—H1*XA*⋯O10*B*^vi^	0.82	2.52	3.194 (4)	140
O1*X*—H1*XA*⋯O26*A*^i^	0.82	2.26	2.811 (4)	125
C4*A*—H4*AB*⋯O13*A*	0.97	2.42	2.992 (2)	117
C11*B*—H11*B*⋯O11*B*^i^	0.93	2.51	3.429 (2)	170
C5*A*—H5*AB*⋯O21*A*^i^	0.97	2.37	3.016 (2)	123
C7*A*—H7*AA*⋯O6*A*	0.97	2.56	3.072 (2)	113
C7*A*—H7*AA*⋯O23*B*	0.97	2.49	3.004 (2)	113
C7*A*—H7*AB*⋯O18*B*^iii^	0.97	2.39	3.278 (2)	152
C17*A*—H17*A*⋯O23*A*^vii^	0.93	2.32	3.235 (3)	167
C19*B*—H19*B*⋯O24*B*^iii^	0.93	2.39	3.255 (3)	155
C21—H21*B*⋯O10*B*^vi^	0.96	2.50	3.236 (3)	133
C1*B*—H1*BA*⋯O26*B*^i^	0.97	2.43	3.085 (3)	125
C3*B*—H3*BB*⋯O15*A*^iii^	0.97	2.59	3.111 (3)	114
C5*B*—H5*BB*⋯O20*B*	0.97	2.60	3.222 (2)	122
C6*B*—H6*BB*⋯O12*B*	0.97	2.51	3.375 (3)	149
C7*B*—H7*BA*⋯O12*B*	0.97	2.56	3.407 (3)	146

Symmetry codes: (i) 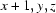; (iii) 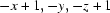; (iv) 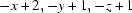; (v) 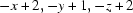; (vi) 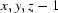; (vii) 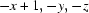.

**Fig. 1.**
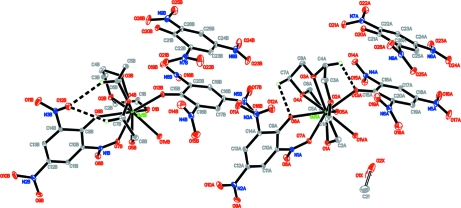
The molecular structure of (I), showing 40% probability displacement ellipsoids. For clarity, only hydrogen atoms involved in intramolecular interactions (shown as dashed lines) were drawn.

**Fig. 2.**
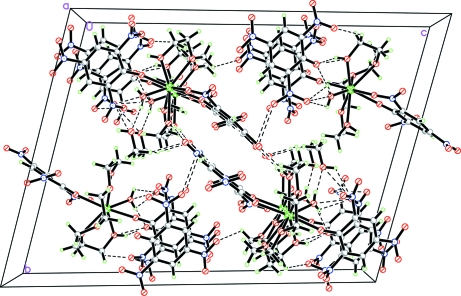
The crystal packing of (I), viewed down the a axis, showing chains along the c axis and stacking of these chains along the a axis. Intermolecular interactions are shown as dashed lines.

**Fig. 3.**
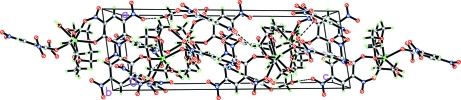
The crystal packing of (I), viewed down the b axis, showing chains along the c axis. Intermolecular interactions are shown as dashed lines.

## supplementary crystallographic information

### Comment

The tetraethylene glycol (EO_4_) molecule was chosen as a ligand, based on the following considerations, (i) it is a flexible ligand and its donor oxygen atoms can be coordinated to Ln^III^ ion in a pentadentate mode, (ii) shielding the encapsulated Ln^III^ ion from further interaction with the surrounding atoms and/or water molecules, and (iii) its terminal hydroxy groups can act not only as oxygen donor atoms but also as hydrogen-bond donors (Kusrini *et al.*, 2008). The lanthanide complexes with the EO_4_ ligand showed the formation of salt-type compounds with molecular formula of [Ln.*X*_n_(EO_4_)], [Ln(OH_2_)*_y_*(EO_4_)][*X*]_n_ or [Ln.*X*_n_.(OH_2_)*_y_*(EO_4_)][*X*], where *X* can be halogen, NO_3_^-^ and SCN^-^ (Rogers, Rollins *et al.*, 1991; Rogers Russel *et al.*, 1991; Rogers & Henry, 1992; Rogers *et al.* 1997; Casellato *et al.*, 1982). Meanwhile, the EO_4_—Ln-Pic complex coordinated to the Ln^III^ ions in the coordination sphere have yet to be described in the literature where Pic = picrate anion. In the title structure, the [Gd(EO_4_)(Pic)_2_(H_2_O)][Pic].0.5CH_3_OH complex was obtained from a solution mixture containing EO_4_, picric acid (HPic) and gadolinium nitrate hexahydrate.

In the title compound (Fig. 1), each gadolinium has nine coordination sites occupied by five oxygen atoms from the tetraethylene glycol (EO_4_) as a pentadentate mode, an oxygen of water molecule, and three oxygen atoms of the two picrate anions as bidentate and monodentate ligands, respectively. Bond lengths and angles have normal values (Allen *et al.*, 1987). The geometry is that of a distorted tricapped trigonal prism. The asymmetric unit of the crystal structure is composed of two crystallographically independent molecules of the complex, (A and B) with two uncoordinated picrate anions and one methanol solvent. The oxygen atom of the methanol molecule is disordered over two positions and the refined site-occupancy factors of the disordered parts are 0.603 (6) and 0.397 (6). The mean plane of the two coordinated mono- and bidentate picrate anions to the Gd^III^ center are almost perpendicular to each other which can be indicated from the dihedral angles of 89.92 (8) and 86.60 (8)° in molecule A and B, respectively. In the crystal structure, molecules are arranged into 2-D infinite networks parallel to the *ac* plane. Because of the presence of many nitro-groups in the structure of the title compound, some of the intermolecular contacts, especially for the oxygen atoms, are shorter than the sum of the van der Waals radii of the relevant atoms (Allen *et al.*, 1998) (Table 1). The crystal structure is stabilized by intramolecular C—H···O (× 4), intermolecular O—H···O (× 13), O—H···N (× 3), C—H···O (× 10) hydrogen bonds (Table 2). π-π interactions between the C15A–C20A (centroid *Cg*1) and C21A–C26A (centroid *Cg*2) rings in the molecule A [*Cg*1···*Cg*2^i^ = 3.8073 (10) Å; symmetry code: (i) *x*, *y*, *z*] and the C15B–C20B (centroid *Cg*3) and C21B–C26B (centroid *Cg*4) rings in the molecule B [*Cg*3···*Cg*4^i^ = 3.9831 (10) Å; symmetry code: (i) 1 + *x*, *y*, *z*] are observed.

### Experimental

Gd(NO_3_)_3_.6H_2_O (0.434 g, 1 mmol), EO_4_ (0.78 g, 4 mmol) and picric acid (0.91 g, 3.97 mmol) were dissolved in 15 ml mixture of acetonitrile-methanol (1:1 *v*/*v*) in a 50 ml beaker. A clear yellow solution was obtained and was then covered by aluminium foil to allow for slow evaporation at room temperature. Yellow crystalline solid was visible after one day with 80% yield. Decomposition point 480.8–499.2 K. Anal. Calc.: C, 29.03; H, 2.32; N, 11.72. Found: C, 30.02; H, 2.20; N, 11.63%.

### Refinement

The hydrogen atoms of the coordinated water molecules, hydroxy groups, and hydrogen atoms bound to the oxygen of the methanol solvent were initially located from the difference Fourier map and refined as riding with the parent atoms after using distance restraints. The rest of the hydrogen atoms were constrained and allowed to ride on the parent atoms. The C—O distances of the disordered methanol were refined with a C—O distance restraint of 1.359 (1) Å. The highest peak is located 0.69 Å from Gd1A and the deepest hole is located 0.49 Å from C21.

### Crystal data

**Table:**

[Gd(C_6_H_2_N_3_O_7_)_2_(C_8_H_18_O_5_)(H_2_O)](C_6_H_2_N_3_O_7_)·0.5CH_4_O	*Z* = 4
*M**_r_* = 1069.83	*F*_000_ = 2136
Triclinic, *P*1	*D*_x_ = 1.951 Mg m^−^^3^
Hall symbol: -P 1	Mo *K*α radiation λ = 0.71073 Å
*a* = 8.1816 (6) Å	Cell parameters from 9911 reflections
*b* = 18.6657 (14) Å	θ = 2.5–37.5º
*c* = 24.9646 (18) Å	µ = 1.94 mm^−^^1^
α = 104.188 (3)º	*T* = 100.0 (1) K
β = 96.445 (4)º	Block, yellow
γ = 95.483 (4)º	0.51 × 0.31 × 0.16 mm
*V* = 3642.7 (5) Å^3^	

### Data collection

**Table:**

Bruker SMART APEXII CCD area-detector diffractometer	38087 independent reflections
Radiation source: fine-focus sealed tube	30033 reflections with *I* > 2σ(*I*)
Monochromator: graphite	*R*_int_ = 0.041
*T* = 100.0(1) K	θ_max_ = 37.5º
φ and ω scans	θ_min_ = 0.9º
Absorption correction: multi-scan(*SADABS*; Bruker, 2005)	*h* = −13→13
*T*_min_ = 0.432, *T*_max_ = 0.736	*k* = −31→31
125488 measured reflections	*l* = −39→42

### Refinement

**Table:**

Refinement on *F*^2^	Secondary atom site location: difference Fourier map
Least-squares matrix: full	Hydrogen site location: inferred from neighbouring sites
*R*[*F*^2^ > 2σ(*F*^2^)] = 0.033	H-atom parameters constrained
*wR*(*F*^2^) = 0.092	*w* = 1/[σ^2^(*F*_o_^2^) + (0.0417*P*)^2^ + 1.2298*P*] where *P* = (*F*_o_^2^ + 2*F*_c_^2^)/3
*S* = 1.08	(Δ/σ)_max_ = 0.004
38087 reflections	Δρ_max_ = 1.66 e Å^−^^3^
1158 parameters	Δρ_min_ = −1.65 e Å^−^^3^
2 restraints	Extinction correction: none
Primary atom site location: structure-invariant direct methods	

### Special details

**Table:**

Experimental. The low-temperature data was collected with the Oxford Cyrosystem Cobra low-temperature attachment.
Geometry. All e.s.d.'s (except the e.s.d. in the dihedral angle between two l.s. planes) are estimated using the full covariance matrix. The cell e.s.d.'s are taken into account individually in the estimation of e.s.d.'s in distances, angles and torsion angles; correlations between e.s.d.'s in cell parameters are only used when they are defined by crystal symmetry. An approximate (isotropic) treatment of cell e.s.d.'s is used for estimating e.s.d.'s involving l.s. planes.
Refinement. Refinement of *F*^2^ against ALL reflections. The weighted *R*-factor *wR* and goodness of fit *S* are based on *F*^2^, conventional *R*-factors *R* are based on *F*, with *F* set to zero for negative *F*^2^. The threshold expression of *F*^2^ > σ(*F*^2^) is used only for calculating *R*-factors(gt) *etc*. and is not relevant to the choice of reflections for refinement. *R*-factors based on *F*^2^ are statistically about twice as large as those based on *F*, and *R*- factors based on ALL data will be even larger.

### Fractional atomic coordinates and isotropic or equivalent isotropic displacement parameters (Å^2^)

**Table:**

	*x*	*y*	*z*	*U*_iso_*/*U*_eq_	Occ. (<1)
Gd1A	0.608042 (9)	0.265676 (4)	0.296852 (3)	0.00868 (2)	
O1A	0.70249 (16)	0.40233 (7)	0.32150 (6)	0.0145 (2)	
H1OA	0.8047	0.4223	0.3428	0.022*	
O2A	0.38621 (15)	0.34928 (7)	0.29427 (6)	0.0135 (2)	
O3A	0.33119 (15)	0.21771 (7)	0.31087 (6)	0.0143 (2)	
O4A	0.59775 (16)	0.15781 (7)	0.33783 (6)	0.0138 (2)	
O5A	0.76342 (16)	0.16250 (7)	0.25563 (6)	0.0134 (2)	
H5OA	0.8025	0.1569	0.2248	0.020*	
O6A	0.59384 (15)	0.31159 (7)	0.39285 (5)	0.0127 (2)	
O7A	0.88396 (15)	0.28346 (8)	0.35329 (6)	0.0150 (2)	
O8A	1.07633 (18)	0.27812 (9)	0.41795 (7)	0.0224 (3)	
O9A	1.15721 (19)	0.48956 (9)	0.57962 (7)	0.0246 (3)	
O10A	0.9409 (2)	0.52591 (10)	0.61570 (7)	0.0309 (4)	
O11A	0.3917 (2)	0.36844 (13)	0.52115 (8)	0.0344 (4)	
O12A	0.36987 (19)	0.40568 (9)	0.44551 (7)	0.0229 (3)	
O13A	0.46255 (16)	0.21694 (7)	0.20835 (5)	0.0139 (2)	
O14A	0.40001 (19)	0.06007 (9)	0.17781 (7)	0.0236 (3)	
O15A	0.6385 (2)	0.03453 (8)	0.15272 (7)	0.0208 (3)	
O16A	0.6813 (3)	0.10092 (10)	−0.02582 (7)	0.0300 (4)	
O17A	0.6941 (3)	0.21908 (10)	−0.02011 (8)	0.0330 (4)	
O18A	0.5510 (2)	0.38656 (8)	0.14289 (7)	0.0265 (3)	
O19A	0.3520 (2)	0.34043 (9)	0.17970 (7)	0.0235 (3)	
N1A	0.94711 (17)	0.29986 (8)	0.40316 (6)	0.0127 (2)	
N2A	1.0056 (2)	0.48615 (9)	0.57909 (7)	0.0204 (3)	
N3A	0.45121 (19)	0.38771 (9)	0.48325 (7)	0.0164 (3)	
N4A	0.52166 (19)	0.07145 (8)	0.15486 (7)	0.0147 (3)	
N5A	0.6605 (2)	0.16457 (10)	−0.00207 (7)	0.0208 (3)	
N6A	0.4659 (2)	0.33349 (9)	0.15173 (7)	0.0178 (3)	
C1A	0.4220 (2)	0.42868 (10)	0.31465 (9)	0.0183 (3)	
H1AA	0.4192	0.4439	0.3545	0.022*	
H1AB	0.3414	0.4529	0.2963	0.022*	
C2A	0.5933 (2)	0.44906 (10)	0.30162 (9)	0.0174 (3)	
H2AA	0.5908	0.4417	0.2617	0.021*	
H2AB	0.6321	0.5010	0.3198	0.021*	
C3A	0.2176 (2)	0.32386 (11)	0.29847 (9)	0.0188 (3)	
H3AA	0.1414	0.3432	0.2745	0.023*	
H3AB	0.1956	0.3411	0.3366	0.023*	
C4A	0.1945 (2)	0.24033 (10)	0.28073 (8)	0.0153 (3)	
H4AA	0.0902	0.2206	0.2898	0.018*	
H4AB	0.1951	0.2227	0.2408	0.018*	
C5A	0.8040 (2)	0.10940 (11)	0.28633 (9)	0.0168 (3)	
H5AA	0.8960	0.1310	0.3158	0.020*	
H5AB	0.8355	0.0656	0.2618	0.020*	
C6A	0.6532 (2)	0.08899 (10)	0.31061 (9)	0.0181 (3)	
H6AA	0.5675	0.0594	0.2814	0.022*	
H6AB	0.6796	0.0603	0.3373	0.022*	
C7A	0.4518 (2)	0.14620 (10)	0.36443 (8)	0.0152 (3)	
H7AA	0.4574	0.1850	0.3987	0.018*	
H7AB	0.4469	0.0985	0.3735	0.018*	
C8A	0.3011 (2)	0.14796 (10)	0.32535 (8)	0.0167 (3)	
H8AA	0.2876	0.1063	0.2924	0.020*	
H8AB	0.2024	0.1461	0.3434	0.020*	
C9A	0.69105 (19)	0.34734 (9)	0.43571 (7)	0.0110 (3)	
C10A	0.8676 (2)	0.34773 (9)	0.44433 (7)	0.0116 (3)	
C11A	0.9715 (2)	0.39089 (10)	0.49124 (7)	0.0135 (3)	
H11A	1.0855	0.3895	0.4948	0.016*	
C12A	0.9006 (2)	0.43587 (10)	0.53250 (7)	0.0145 (3)	
C13A	0.7286 (2)	0.43369 (10)	0.53063 (8)	0.0144 (3)	
H13A	0.6824	0.4610	0.5603	0.017*	
C14A	0.6309 (2)	0.39024 (9)	0.48405 (7)	0.0123 (3)	
C15A	0.4911 (2)	0.20371 (9)	0.15823 (7)	0.0120 (3)	
C16A	0.5279 (2)	0.13263 (9)	0.12793 (7)	0.0119 (3)	
O26B	0.0053 (2)	0.05816 (10)	0.69472 (7)	0.0287 (4)	
O25B	0.0163 (2)	−0.02844 (9)	0.62066 (8)	0.0285 (3)	
C17A	0.5823 (2)	0.11839 (10)	0.07656 (7)	0.0137 (3)	
H17A	0.6083	0.0715	0.0596	0.016*	
C18A	0.5970 (2)	0.17664 (10)	0.05095 (8)	0.0154 (3)	
C19A	0.5578 (2)	0.24678 (10)	0.07571 (8)	0.0165 (3)	
H19A	0.5684	0.2851	0.0580	0.020*	
C20A	0.5029 (2)	0.25867 (9)	0.12696 (8)	0.0138 (3)	
O20A	−0.08406 (16)	0.19331 (7)	0.17167 (6)	0.0145 (2)	
O21A	0.02614 (19)	0.07122 (8)	0.19759 (6)	0.0192 (3)	
O22A	0.0011 (2)	−0.02114 (8)	0.12384 (7)	0.0220 (3)	
O23A	0.2676 (2)	0.03397 (9)	−0.02551 (7)	0.0271 (3)	
O24A	0.2653 (2)	0.14258 (9)	−0.04213 (7)	0.0244 (3)	
O25A	0.10238 (18)	0.33937 (8)	0.08951 (7)	0.0201 (3)	
O26A	−0.1005 (2)	0.31757 (8)	0.13400 (7)	0.0239 (3)	
N7A	0.01859 (19)	0.04607 (9)	0.14720 (7)	0.0148 (3)	
N8A	0.2310 (2)	0.09754 (10)	−0.01506 (7)	0.0176 (3)	
N9A	0.01088 (19)	0.29658 (9)	0.10700 (7)	0.0148 (3)	
C21A	−0.01043 (19)	0.17214 (9)	0.13013 (7)	0.0118 (3)	
C22A	0.0393 (2)	0.09818 (10)	0.11261 (7)	0.0129 (3)	
C23A	0.1114 (2)	0.07294 (10)	0.06538 (8)	0.0140 (3)	
H23A	0.1361	0.0242	0.0554	0.017*	
C24A	0.1464 (2)	0.12191 (10)	0.03279 (8)	0.0145 (3)	
C25A	0.1119 (2)	0.19470 (10)	0.04735 (8)	0.0140 (3)	
H25A	0.1395	0.2272	0.0259	0.017*	
C26A	0.0361 (2)	0.21845 (9)	0.09399 (8)	0.0128 (3)	
O1WA	0.78489 (17)	0.30413 (7)	0.23642 (6)	0.0155 (2)	
H1WA	0.7288	0.3225	0.2135	0.023*	
H2WA	0.8226	0.2664	0.2174	0.023*	
Gd1B	0.623445 (9)	0.270023 (4)	0.795291 (3)	0.00896 (2)	
O1B	0.76865 (16)	0.16094 (7)	0.75866 (6)	0.0140 (2)	
H1OB	0.7983	0.1574	0.7271	0.021*	
O2B	0.62109 (17)	0.17222 (7)	0.84744 (6)	0.0157 (2)	
O3B	0.35138 (16)	0.20964 (8)	0.80006 (6)	0.0170 (3)	
O4B	0.39681 (15)	0.35279 (7)	0.79379 (6)	0.0137 (2)	
O5B	0.71578 (16)	0.40701 (7)	0.81105 (6)	0.0142 (2)	
H5OB	0.7744	0.4327	0.8421	0.021*	
O6B	0.59521 (15)	0.32557 (7)	0.88968 (5)	0.0124 (2)	
O7B	0.89350 (16)	0.29763 (8)	0.85595 (6)	0.0152 (2)	
O8B	1.07183 (17)	0.28653 (9)	0.92279 (6)	0.0198 (3)	
O9B	1.13002 (17)	0.49605 (8)	1.08765 (6)	0.0183 (3)	
O10B	0.9107 (2)	0.50501 (9)	1.12945 (7)	0.0255 (3)	
O11B	0.37097 (19)	0.37478 (13)	1.01842 (8)	0.0332 (4)	
O12B	0.36282 (17)	0.41048 (9)	0.94234 (6)	0.0194 (3)	
O13B	0.49420 (16)	0.23621 (7)	0.70484 (5)	0.0147 (2)	
O14B	0.4135 (2)	0.35851 (9)	0.66194 (8)	0.0296 (4)	
O15B	0.6582 (2)	0.39089 (9)	0.64435 (9)	0.0350 (4)	
O16B	0.7352 (2)	0.20034 (11)	0.47319 (7)	0.0300 (4)	
O17B	0.6716 (3)	0.08345 (10)	0.46982 (7)	0.0339 (4)	
O18B	0.6098 (2)	0.03455 (8)	0.65242 (7)	0.0220 (3)	
O19B	0.40166 (19)	0.08514 (9)	0.68585 (7)	0.0225 (3)	
N1B	0.94693 (17)	0.31047 (8)	0.90670 (6)	0.0128 (2)	
N2B	0.9795 (2)	0.48073 (9)	1.08858 (7)	0.0156 (3)	
N3B	0.43708 (19)	0.39219 (9)	0.98113 (7)	0.0149 (3)	
N4B	0.5420 (2)	0.34383 (9)	0.64440 (7)	0.0185 (3)	
N5B	0.6820 (2)	0.15013 (11)	0.49316 (7)	0.0214 (3)	
N6B	0.5149 (2)	0.08225 (9)	0.65742 (7)	0.0155 (3)	
C1B	0.7688 (3)	0.09786 (11)	0.78241 (9)	0.0202 (4)	
H1BA	0.8706	0.0757	0.7780	0.024*	
H1BB	0.6758	0.0604	0.7641	0.024*	
C2B	0.7550 (3)	0.12655 (11)	0.84265 (9)	0.0202 (3)	
H2BA	0.7324	0.0853	0.8591	0.024*	
H2BB	0.8582	0.1559	0.8623	0.024*	
C3B	0.4660 (3)	0.13103 (12)	0.84965 (10)	0.0240 (4)	
H3BA	0.4708	0.1156	0.8841	0.029*	
H3BB	0.4428	0.0868	0.8186	0.029*	
C4B	0.3342 (3)	0.17916 (13)	0.84669 (10)	0.0235 (4)	
H4BA	0.2260	0.1503	0.8418	0.028*	
H4BB	0.3459	0.2188	0.8808	0.028*	
C5B	0.2127 (2)	0.24281 (11)	0.77987 (8)	0.0164 (3)	
H5BA	0.1109	0.2207	0.7891	0.020*	
H5BB	0.2029	0.2335	0.7396	0.020*	
C6B	0.2388 (2)	0.32506 (11)	0.80617 (9)	0.0182 (3)	
H6BA	0.1510	0.3488	0.7909	0.022*	
H6BB	0.2393	0.3350	0.8462	0.022*	
C7B	0.4356 (2)	0.43206 (10)	0.81514 (9)	0.0170 (3)	
H7BA	0.4470	0.4459	0.8556	0.020*	
H7BB	0.3487	0.4572	0.8008	0.020*	
C8B	0.5965 (2)	0.45294 (10)	0.79583 (8)	0.0156 (3)	
H8BA	0.5800	0.4456	0.7557	0.019*	
H8BB	0.6365	0.5050	0.8131	0.019*	
C9B	0.68495 (19)	0.35609 (9)	0.93546 (7)	0.0107 (3)	
C10B	0.8610 (2)	0.35562 (9)	0.94724 (7)	0.0116 (3)	
C11B	0.9585 (2)	0.39483 (9)	0.99652 (7)	0.0126 (3)	
H11B	1.0729	0.3947	1.0011	0.015*	
C12B	0.8811 (2)	0.43424 (10)	1.03869 (7)	0.0132 (3)	
C13B	0.7086 (2)	0.42982 (10)	1.03446 (7)	0.0135 (3)	
H13B	0.6574	0.4511	1.0649	0.016*	
C14B	0.6169 (2)	0.39315 (9)	0.98415 (7)	0.0121 (3)	
C15B	0.5251 (2)	0.21481 (9)	0.65524 (7)	0.0123 (3)	
C16B	0.5606 (2)	0.26544 (10)	0.62181 (8)	0.0143 (3)	
C17B	0.6144 (2)	0.24619 (10)	0.57067 (8)	0.0165 (3)	
H17B	0.6421	0.2821	0.5520	0.020*	
C18B	0.6263 (2)	0.17137 (11)	0.54758 (8)	0.0162 (3)	
C19B	0.5903 (2)	0.11747 (10)	0.57550 (8)	0.0153 (3)	
H19B	0.5994	0.0677	0.5597	0.018*	
C20B	0.5405 (2)	0.13945 (9)	0.62743 (7)	0.0133 (3)	
O20B	−0.07838 (16)	0.18632 (7)	0.67524 (6)	0.0144 (2)	
O21B	0.0602 (2)	0.32506 (8)	0.67484 (7)	0.0252 (3)	
O22B	0.0310 (3)	0.33386 (10)	0.59029 (8)	0.0379 (5)	
O23B	0.2717 (2)	0.15015 (10)	0.46334 (7)	0.0295 (4)	
O24B	0.2570 (3)	0.03689 (11)	0.47143 (8)	0.0322 (4)	
N7B	0.0490 (2)	0.29762 (9)	0.62461 (8)	0.0197 (3)	
N8B	0.2323 (2)	0.10193 (11)	0.48694 (8)	0.0210 (3)	
N9B	0.0183 (2)	0.03712 (9)	0.64518 (7)	0.0176 (3)	
C21B	0.0047 (2)	0.16787 (10)	0.63562 (7)	0.0125 (3)	
C22B	0.0628 (2)	0.21878 (10)	0.60433 (8)	0.0140 (3)	
C23B	0.1300 (2)	0.19784 (11)	0.55588 (8)	0.0165 (3)	
H23B	0.1586	0.2325	0.5362	0.020*	
C24B	0.1545 (2)	0.12411 (11)	0.53678 (8)	0.0162 (3)	
C25B	0.1153 (2)	0.07156 (11)	0.56596 (8)	0.0162 (3)	
H25B	0.1357	0.0225	0.5531	0.019*	
C26B	0.0457 (2)	0.09360 (10)	0.61429 (8)	0.0142 (3)	
O1WB	0.82835 (17)	0.30080 (7)	0.74100 (6)	0.0173 (3)	
H1WB	0.8252	0.3356	0.7236	0.026*	
H2WB	0.8524	0.2637	0.7161	0.026*	
C21	0.9877 (4)	0.50655 (12)	0.25975 (12)	0.0486 (8)	
H21A	1.0939	0.5151	0.2825	0.073*	0.603 (6)
H21B	0.9901	0.5357	0.2330	0.073*	0.603 (6)
H21C	0.9041	0.5208	0.2830	0.073*	0.603 (6)
H21D	1.0729	0.4871	0.2796	0.073*	0.397 (6)
H21E	1.0365	0.5338	0.2363	0.073*	0.397 (6)
H21F	0.9330	0.5391	0.2860	0.073*	0.397 (6)
O1X	0.9553 (7)	0.43300 (12)	0.23294 (15)	0.0384 (9)	0.603 (6)
H1XA	0.9424	0.4281	0.1991	0.058*	0.603 (6)
O2X	0.8802 (7)	0.4470 (2)	0.2285 (2)	0.0384 (9)	0.397 (6)
H1XB	0.9278	0.4097	0.2230	0.058*	0.397 (6)

### Atomic displacement parameters (Å^2^)

**Table:**

	*U*^11^	*U*^22^	*U*^33^	*U*^12^	*U*^13^	*U*^23^
Gd1A	0.00928 (3)	0.00890 (3)	0.00746 (4)	0.00089 (2)	0.00152 (2)	0.00129 (2)
O1A	0.0159 (5)	0.0117 (5)	0.0139 (6)	0.0005 (4)	−0.0007 (4)	0.0013 (4)
O2A	0.0133 (5)	0.0126 (5)	0.0144 (6)	0.0030 (4)	0.0028 (4)	0.0023 (4)
O3A	0.0109 (5)	0.0158 (5)	0.0167 (6)	−0.0007 (4)	0.0004 (4)	0.0068 (5)
O4A	0.0156 (5)	0.0120 (5)	0.0165 (6)	0.0031 (4)	0.0086 (4)	0.0058 (4)
O5A	0.0162 (5)	0.0124 (5)	0.0136 (6)	0.0043 (4)	0.0056 (4)	0.0046 (4)
O6A	0.0115 (5)	0.0158 (5)	0.0085 (5)	−0.0007 (4)	0.0007 (4)	−0.0001 (4)
O7A	0.0120 (5)	0.0211 (6)	0.0098 (6)	0.0016 (4)	0.0007 (4)	0.0008 (5)
O8A	0.0158 (6)	0.0304 (8)	0.0207 (7)	0.0106 (5)	−0.0005 (5)	0.0046 (6)
O9A	0.0236 (7)	0.0241 (7)	0.0213 (8)	−0.0081 (6)	−0.0079 (5)	0.0060 (6)
O10A	0.0424 (10)	0.0256 (8)	0.0157 (7)	−0.0028 (7)	−0.0006 (7)	−0.0069 (6)
O11A	0.0209 (7)	0.0640 (13)	0.0272 (9)	0.0093 (8)	0.0119 (6)	0.0233 (9)
O12A	0.0203 (6)	0.0293 (8)	0.0209 (7)	0.0096 (6)	0.0005 (5)	0.0083 (6)
O13A	0.0145 (5)	0.0178 (6)	0.0083 (5)	0.0018 (4)	0.0014 (4)	0.0012 (4)
O14A	0.0225 (6)	0.0266 (7)	0.0247 (8)	−0.0016 (6)	0.0078 (6)	0.0119 (6)
O15A	0.0280 (7)	0.0145 (6)	0.0216 (7)	0.0075 (5)	0.0053 (6)	0.0053 (5)
O16A	0.0508 (11)	0.0251 (8)	0.0191 (8)	0.0156 (7)	0.0177 (7)	0.0056 (6)
O17A	0.0573 (12)	0.0258 (8)	0.0220 (8)	0.0049 (8)	0.0190 (8)	0.0116 (7)
O18A	0.0382 (9)	0.0136 (6)	0.0271 (8)	0.0030 (6)	0.0040 (7)	0.0042 (6)
O19A	0.0295 (7)	0.0247 (7)	0.0174 (7)	0.0130 (6)	0.0054 (6)	0.0031 (6)
N1A	0.0112 (5)	0.0149 (6)	0.0114 (6)	0.0021 (4)	0.0012 (4)	0.0026 (5)
N2A	0.0284 (8)	0.0160 (7)	0.0138 (7)	−0.0052 (6)	−0.0041 (6)	0.0041 (6)
N3A	0.0152 (6)	0.0191 (7)	0.0147 (7)	0.0051 (5)	0.0039 (5)	0.0021 (6)
N4A	0.0184 (6)	0.0125 (6)	0.0122 (7)	−0.0008 (5)	0.0016 (5)	0.0025 (5)
N5A	0.0301 (8)	0.0221 (8)	0.0122 (7)	0.0045 (6)	0.0067 (6)	0.0059 (6)
N6A	0.0244 (7)	0.0152 (7)	0.0131 (7)	0.0066 (5)	−0.0004 (6)	0.0023 (5)
C1A	0.0213 (8)	0.0116 (7)	0.0233 (9)	0.0061 (6)	0.0062 (7)	0.0042 (6)
C2A	0.0210 (8)	0.0126 (7)	0.0187 (9)	0.0017 (6)	0.0024 (6)	0.0047 (6)
C3A	0.0119 (6)	0.0195 (8)	0.0263 (10)	0.0048 (6)	0.0062 (6)	0.0057 (7)
C4A	0.0099 (6)	0.0196 (8)	0.0159 (8)	0.0019 (5)	0.0015 (5)	0.0039 (6)
C5A	0.0172 (7)	0.0176 (7)	0.0208 (9)	0.0069 (6)	0.0077 (6)	0.0107 (7)
C6A	0.0193 (7)	0.0140 (7)	0.0245 (10)	0.0038 (6)	0.0083 (7)	0.0086 (7)
C7A	0.0176 (7)	0.0148 (7)	0.0157 (8)	0.0018 (6)	0.0079 (6)	0.0063 (6)
C8A	0.0156 (7)	0.0151 (7)	0.0194 (9)	−0.0019 (6)	0.0046 (6)	0.0048 (6)
C9A	0.0116 (6)	0.0111 (6)	0.0101 (7)	0.0011 (5)	0.0010 (5)	0.0028 (5)
C10A	0.0127 (6)	0.0124 (6)	0.0089 (7)	0.0007 (5)	0.0009 (5)	0.0020 (5)
C11A	0.0136 (6)	0.0149 (7)	0.0115 (7)	−0.0017 (5)	−0.0008 (5)	0.0049 (6)
C12A	0.0190 (7)	0.0121 (7)	0.0099 (7)	−0.0022 (5)	−0.0021 (5)	0.0014 (5)
C13A	0.0193 (7)	0.0125 (7)	0.0103 (7)	0.0014 (5)	0.0003 (6)	0.0017 (5)
C14A	0.0141 (6)	0.0126 (6)	0.0097 (7)	0.0021 (5)	0.0019 (5)	0.0014 (5)
C15A	0.0118 (6)	0.0138 (7)	0.0092 (7)	0.0013 (5)	0.0008 (5)	0.0014 (5)
C16A	0.0140 (6)	0.0107 (6)	0.0113 (7)	0.0010 (5)	0.0020 (5)	0.0035 (5)
O26B	0.0447 (10)	0.0307 (8)	0.0210 (8)	0.0169 (7)	0.0188 (7)	0.0151 (7)
O25B	0.0439 (10)	0.0182 (7)	0.0265 (9)	0.0086 (6)	0.0066 (7)	0.0091 (6)
C17A	0.0179 (7)	0.0127 (7)	0.0104 (7)	0.0038 (5)	0.0034 (5)	0.0018 (5)
C18A	0.0207 (7)	0.0155 (7)	0.0104 (7)	0.0024 (6)	0.0033 (6)	0.0037 (6)
C19A	0.0234 (8)	0.0146 (7)	0.0118 (8)	0.0016 (6)	0.0023 (6)	0.0044 (6)
C20A	0.0184 (7)	0.0116 (6)	0.0114 (7)	0.0034 (5)	0.0013 (5)	0.0026 (5)
O20A	0.0161 (5)	0.0162 (5)	0.0125 (6)	0.0044 (4)	0.0062 (4)	0.0033 (4)
O21A	0.0251 (6)	0.0221 (6)	0.0136 (6)	0.0077 (5)	0.0069 (5)	0.0068 (5)
O22A	0.0292 (7)	0.0140 (6)	0.0233 (8)	0.0045 (5)	0.0035 (6)	0.0056 (5)
O23A	0.0381 (9)	0.0251 (7)	0.0219 (8)	0.0170 (7)	0.0140 (7)	0.0039 (6)
O24A	0.0326 (8)	0.0267 (7)	0.0181 (7)	0.0068 (6)	0.0143 (6)	0.0078 (6)
O25A	0.0238 (6)	0.0172 (6)	0.0233 (7)	0.0025 (5)	0.0085 (5)	0.0103 (5)
O26A	0.0315 (7)	0.0194 (6)	0.0270 (8)	0.0111 (6)	0.0177 (6)	0.0084 (6)
N7A	0.0148 (6)	0.0153 (6)	0.0167 (7)	0.0047 (5)	0.0047 (5)	0.0068 (5)
N8A	0.0198 (7)	0.0213 (7)	0.0123 (7)	0.0068 (6)	0.0059 (5)	0.0019 (6)
N9A	0.0176 (6)	0.0145 (6)	0.0136 (7)	0.0037 (5)	0.0041 (5)	0.0045 (5)
C21A	0.0107 (6)	0.0137 (6)	0.0112 (7)	0.0018 (5)	0.0026 (5)	0.0029 (5)
C22A	0.0142 (6)	0.0136 (7)	0.0117 (7)	0.0033 (5)	0.0035 (5)	0.0034 (6)
C23A	0.0146 (6)	0.0160 (7)	0.0118 (7)	0.0049 (5)	0.0040 (5)	0.0022 (6)
C24A	0.0159 (7)	0.0166 (7)	0.0115 (7)	0.0047 (5)	0.0045 (5)	0.0023 (6)
C25A	0.0150 (6)	0.0165 (7)	0.0117 (7)	0.0036 (5)	0.0043 (5)	0.0042 (6)
C26A	0.0154 (6)	0.0108 (6)	0.0130 (7)	0.0027 (5)	0.0051 (5)	0.0026 (5)
O1WA	0.0189 (6)	0.0133 (5)	0.0150 (6)	0.0006 (4)	0.0078 (5)	0.0031 (5)
Gd1B	0.00998 (3)	0.00894 (3)	0.00780 (4)	0.00133 (2)	0.00178 (2)	0.00161 (2)
O1B	0.0186 (5)	0.0129 (5)	0.0131 (6)	0.0052 (4)	0.0079 (4)	0.0048 (4)
O2B	0.0194 (6)	0.0143 (5)	0.0176 (6)	0.0056 (4)	0.0088 (5)	0.0079 (5)
O3B	0.0131 (5)	0.0220 (6)	0.0179 (7)	−0.0014 (4)	0.0018 (4)	0.0103 (5)
O4B	0.0122 (5)	0.0132 (5)	0.0158 (6)	0.0023 (4)	0.0033 (4)	0.0028 (4)
O5B	0.0141 (5)	0.0105 (5)	0.0164 (6)	0.0018 (4)	0.0012 (4)	0.0008 (4)
O6B	0.0120 (5)	0.0152 (5)	0.0086 (5)	0.0007 (4)	0.0009 (4)	0.0009 (4)
O7B	0.0126 (5)	0.0227 (6)	0.0093 (6)	0.0025 (4)	0.0018 (4)	0.0022 (5)
O8B	0.0145 (5)	0.0257 (7)	0.0187 (7)	0.0095 (5)	0.0002 (5)	0.0034 (5)
O9B	0.0174 (6)	0.0166 (6)	0.0180 (7)	−0.0030 (5)	−0.0038 (5)	0.0038 (5)
O10B	0.0275 (7)	0.0297 (8)	0.0127 (7)	0.0056 (6)	0.0005 (5)	−0.0065 (6)
O11B	0.0162 (6)	0.0674 (13)	0.0239 (8)	0.0080 (7)	0.0086 (6)	0.0233 (9)
O12B	0.0174 (6)	0.0248 (7)	0.0174 (7)	0.0079 (5)	0.0012 (5)	0.0066 (5)
O13B	0.0164 (5)	0.0178 (6)	0.0091 (5)	0.0035 (4)	0.0023 (4)	0.0012 (4)
O14B	0.0292 (8)	0.0201 (7)	0.0386 (10)	0.0091 (6)	0.0072 (7)	0.0026 (7)
O15B	0.0457 (10)	0.0177 (7)	0.0405 (11)	−0.0059 (7)	0.0152 (8)	0.0049 (7)
O16B	0.0370 (9)	0.0382 (9)	0.0189 (8)	0.0030 (7)	0.0121 (7)	0.0120 (7)
O17B	0.0593 (12)	0.0299 (9)	0.0176 (8)	0.0200 (8)	0.0163 (8)	0.0057 (7)
O18B	0.0315 (7)	0.0131 (6)	0.0230 (7)	0.0065 (5)	0.0054 (6)	0.0055 (5)
O19B	0.0255 (7)	0.0226 (7)	0.0216 (7)	0.0003 (5)	0.0089 (6)	0.0081 (6)
N1B	0.0116 (5)	0.0156 (6)	0.0111 (6)	0.0013 (5)	0.0022 (4)	0.0031 (5)
N2B	0.0207 (7)	0.0120 (6)	0.0121 (7)	0.0017 (5)	−0.0019 (5)	0.0012 (5)
N3B	0.0136 (6)	0.0190 (7)	0.0118 (7)	0.0046 (5)	0.0028 (5)	0.0021 (5)
N4B	0.0273 (8)	0.0132 (6)	0.0149 (7)	0.0025 (6)	0.0006 (6)	0.0043 (5)
N5B	0.0261 (8)	0.0280 (9)	0.0125 (7)	0.0092 (7)	0.0056 (6)	0.0061 (6)
N6B	0.0206 (6)	0.0122 (6)	0.0131 (7)	−0.0005 (5)	0.0012 (5)	0.0035 (5)
C1B	0.0291 (9)	0.0188 (8)	0.0192 (9)	0.0118 (7)	0.0119 (7)	0.0098 (7)
C2B	0.0239 (8)	0.0206 (8)	0.0200 (9)	0.0082 (7)	0.0066 (7)	0.0091 (7)
C3B	0.0229 (8)	0.0246 (9)	0.0299 (11)	−0.0001 (7)	0.0067 (8)	0.0172 (8)
C4B	0.0223 (8)	0.0271 (10)	0.0270 (11)	0.0038 (7)	0.0122 (8)	0.0140 (8)
C5B	0.0109 (6)	0.0208 (8)	0.0164 (8)	−0.0007 (6)	0.0007 (5)	0.0043 (6)
C6B	0.0107 (6)	0.0211 (8)	0.0216 (9)	0.0011 (6)	0.0040 (6)	0.0025 (7)
C7B	0.0164 (7)	0.0129 (7)	0.0226 (9)	0.0056 (5)	0.0053 (6)	0.0040 (6)
C8B	0.0181 (7)	0.0121 (7)	0.0176 (8)	0.0030 (5)	0.0043 (6)	0.0045 (6)
C9B	0.0116 (6)	0.0100 (6)	0.0104 (7)	0.0011 (5)	0.0020 (5)	0.0028 (5)
C10B	0.0118 (6)	0.0124 (6)	0.0103 (7)	0.0008 (5)	0.0018 (5)	0.0022 (5)
C11B	0.0124 (6)	0.0130 (6)	0.0123 (7)	0.0004 (5)	0.0008 (5)	0.0038 (5)
C12B	0.0148 (6)	0.0130 (7)	0.0104 (7)	0.0010 (5)	−0.0017 (5)	0.0022 (5)
C13B	0.0166 (7)	0.0135 (7)	0.0095 (7)	0.0035 (5)	0.0008 (5)	0.0011 (5)
C14B	0.0124 (6)	0.0130 (6)	0.0102 (7)	0.0021 (5)	0.0020 (5)	0.0009 (5)
C15B	0.0133 (6)	0.0134 (7)	0.0097 (7)	0.0020 (5)	0.0001 (5)	0.0024 (5)
C16B	0.0187 (7)	0.0128 (7)	0.0106 (7)	0.0020 (5)	0.0008 (5)	0.0018 (6)
C17B	0.0209 (7)	0.0164 (7)	0.0128 (8)	0.0013 (6)	0.0031 (6)	0.0050 (6)
C18B	0.0198 (7)	0.0195 (8)	0.0098 (7)	0.0050 (6)	0.0029 (6)	0.0034 (6)
C19B	0.0195 (7)	0.0155 (7)	0.0106 (7)	0.0055 (6)	0.0021 (6)	0.0016 (6)
C20B	0.0176 (7)	0.0113 (6)	0.0104 (7)	0.0021 (5)	0.0015 (5)	0.0018 (5)
O20B	0.0155 (5)	0.0156 (5)	0.0133 (6)	0.0032 (4)	0.0062 (4)	0.0036 (4)
O21B	0.0370 (8)	0.0183 (6)	0.0198 (7)	−0.0018 (6)	0.0134 (6)	0.0017 (5)
O22B	0.0700 (14)	0.0245 (8)	0.0275 (9)	0.0178 (9)	0.0126 (9)	0.0154 (7)
O23B	0.0364 (9)	0.0365 (9)	0.0220 (8)	0.0075 (7)	0.0175 (7)	0.0125 (7)
O24B	0.0472 (10)	0.0344 (9)	0.0222 (8)	0.0225 (8)	0.0188 (7)	0.0080 (7)
N7B	0.0229 (7)	0.0174 (7)	0.0224 (8)	0.0056 (6)	0.0106 (6)	0.0071 (6)
N8B	0.0222 (7)	0.0287 (9)	0.0151 (8)	0.0096 (6)	0.0085 (6)	0.0064 (6)
N9B	0.0202 (7)	0.0191 (7)	0.0179 (8)	0.0081 (5)	0.0068 (6)	0.0091 (6)
C21B	0.0122 (6)	0.0149 (7)	0.0105 (7)	0.0018 (5)	0.0020 (5)	0.0031 (5)
C22B	0.0163 (7)	0.0148 (7)	0.0132 (7)	0.0045 (5)	0.0050 (6)	0.0059 (6)
C23B	0.0160 (7)	0.0223 (8)	0.0145 (8)	0.0055 (6)	0.0059 (6)	0.0079 (7)
C24B	0.0169 (7)	0.0225 (8)	0.0112 (7)	0.0058 (6)	0.0053 (6)	0.0054 (6)
C25B	0.0176 (7)	0.0195 (8)	0.0139 (8)	0.0075 (6)	0.0060 (6)	0.0052 (6)
C26B	0.0164 (7)	0.0164 (7)	0.0115 (7)	0.0043 (5)	0.0046 (5)	0.0045 (6)
O1WB	0.0226 (6)	0.0127 (5)	0.0171 (6)	−0.0002 (5)	0.0111 (5)	0.0019 (5)
C21	0.0408 (15)	0.0489 (17)	0.056 (2)	−0.0168 (13)	−0.0103 (13)	0.0297 (15)
O1X	0.078 (3)	0.0113 (10)	0.0331 (13)	0.0103 (12)	0.0302 (17)	0.0080 (9)
O2X	0.078 (3)	0.0113 (10)	0.0331 (13)	0.0103 (12)	0.0302 (17)	0.0080 (9)

### Geometric parameters (Å, °)

**Table:**

Gd1A—O13A	2.3115 (13)	Gd1B—O3B	2.4259 (12)
Gd1A—O6A	2.3593 (13)	Gd1B—O7B	2.4676 (13)
Gd1A—O1WA	2.3889 (12)	Gd1B—O1B	2.4874 (13)
Gd1A—O3A	2.4492 (12)	Gd1B—O2B	2.4884 (13)
Gd1A—O4A	2.4708 (12)	Gd1B—O5B	2.5169 (12)
Gd1A—O7A	2.4708 (13)	Gd1B—O4B	2.5271 (13)
Gd1A—O5A	2.4848 (13)	O1B—C1B	1.442 (2)
Gd1A—O1A	2.4958 (13)	O1B—H1OB	0.8384
Gd1A—O2A	2.5096 (13)	O2B—C3B	1.434 (2)
O1A—C2A	1.438 (2)	O2B—C2B	1.449 (2)
O1A—H1OA	0.9300	O3B—C4B	1.430 (2)
O2A—C1A	1.434 (2)	O3B—C5B	1.443 (2)
O2A—C3A	1.439 (2)	O4B—C7B	1.435 (2)
O3A—C4A	1.432 (2)	O4B—C6B	1.441 (2)
O3A—C8A	1.441 (2)	O5B—C8B	1.438 (2)
O4A—C6A	1.439 (2)	O5B—H5OB	0.8661
O4A—C7A	1.453 (2)	O6B—C9B	1.258 (2)
O5A—C5A	1.435 (2)	O7B—N1B	1.249 (2)
O5A—H5OA	0.8530	O8B—N1B	1.220 (2)
O6A—C9A	1.257 (2)	O9B—N2B	1.241 (2)
O7A—N1A	1.245 (2)	O10B—N2B	1.230 (2)
O8A—N1A	1.219 (2)	O11B—N3B	1.222 (2)
O9A—N2A	1.235 (2)	O12B—N3B	1.222 (2)
O10A—N2A	1.232 (3)	O13B—C15B	1.266 (2)
O11A—N3A	1.224 (2)	O14B—N4B	1.213 (3)
O12A—N3A	1.225 (2)	O15B—N4B	1.232 (2)
O13A—C15A	1.267 (2)	O16B—N5B	1.232 (2)
O14A—N4A	1.229 (2)	O17B—N5B	1.228 (3)
O15A—N4A	1.229 (2)	O18B—N6B	1.229 (2)
O16A—N5A	1.228 (2)	O19B—N6B	1.226 (2)
O17A—N5A	1.230 (2)	N1B—C10B	1.444 (2)
O18A—N6A	1.235 (2)	N2B—C12B	1.437 (2)
O19A—N6A	1.224 (2)	N3B—C14B	1.463 (2)
N1A—C10A	1.446 (2)	N4B—C16B	1.463 (2)
N2A—C12A	1.435 (2)	N5B—C18B	1.456 (3)
N3A—C14A	1.464 (2)	N6B—C20B	1.459 (2)
N4A—C16A	1.460 (2)	C1B—C2B	1.489 (3)
N5A—C18A	1.450 (3)	C1B—H1BA	0.9700
N6A—C20A	1.457 (2)	C1B—H1BB	0.9700
C1A—C2A	1.509 (3)	C2B—H2BA	0.9700
C1A—H1AA	0.9700	C2B—H2BB	0.9700
C1A—H1AB	0.9700	C3B—C4B	1.474 (3)
C2A—H2AA	0.9700	C3B—H3BA	0.9700
C2A—H2AB	0.9700	C3B—H3BB	0.9700
C3A—C4A	1.500 (3)	C4B—H4BA	0.9700
C3A—H3AA	0.9700	C4B—H4BB	0.9700
C3A—H3AB	0.9700	C5B—C6B	1.498 (3)
C4A—H4AA	0.9700	C5B—H5BA	0.9700
C4A—H4AB	0.9700	C5B—H5BB	0.9700
C5A—C6A	1.492 (3)	C6B—H6BA	0.9700
C5A—H5AA	0.9700	C6B—H6BB	0.9700
C5A—H5AB	0.9700	C7B—C8B	1.501 (3)
C6A—H6AA	0.9700	C7B—H7BA	0.9700
C6A—H6AB	0.9700	C7B—H7BB	0.9700
C7A—C8A	1.492 (3)	C8B—H8BA	0.9700
C7A—H7AA	0.9700	C8B—H8BB	0.9700
C7A—H7AB	0.9700	C9B—C10B	1.438 (2)
C8A—H8AA	0.9700	C9B—C14B	1.442 (2)
C8A—H8AB	0.9700	C10B—C11B	1.383 (2)
C9A—C10A	1.435 (2)	C11B—C12B	1.382 (3)
C9A—C14A	1.440 (3)	C11B—H11B	0.9300
C10A—C11A	1.388 (2)	C12B—C13B	1.397 (2)
C11A—C12A	1.381 (3)	C13B—C14B	1.370 (2)
C11A—H11A	0.9300	C13B—H13B	0.9300
C12A—C13A	1.399 (3)	C15B—C20B	1.432 (2)
C13A—C14A	1.362 (2)	C15B—C16B	1.436 (2)
C13A—H13A	0.9300	C16B—C17B	1.372 (3)
C15A—C16A	1.435 (2)	C17B—C18B	1.391 (3)
C15A—C20A	1.436 (2)	C17B—H17B	0.9300
C16A—C17A	1.377 (2)	C18B—C19B	1.385 (3)
O26B—N9B	1.223 (2)	C19B—C20B	1.380 (3)
O25B—N9B	1.224 (2)	C19B—H19B	0.9300
C17A—C18A	1.392 (2)	O20B—C21B	1.260 (2)
C17A—H17A	0.9300	O21B—N7B	1.222 (2)
C18A—C19A	1.388 (3)	O22B—N7B	1.220 (2)
C19A—C20A	1.377 (3)	O23B—N8B	1.228 (2)
C19A—H19A	0.9300	O24B—N8B	1.224 (3)
O20A—C21A	1.257 (2)	N7B—C22B	1.454 (3)
O21A—N7A	1.222 (2)	N8B—C24B	1.449 (3)
O22A—N7A	1.234 (2)	N9B—C26B	1.466 (2)
O23A—N8A	1.225 (2)	C21B—C26B	1.444 (3)
O24A—N8A	1.232 (2)	C21B—C22B	1.449 (2)
O25A—N9A	1.2325 (19)	C22B—C23B	1.370 (3)
O26A—N9A	1.231 (2)	C23B—C24B	1.383 (3)
N7A—C22A	1.461 (2)	C23B—H23B	0.9300
N8A—C24A	1.446 (2)	C24B—C25B	1.392 (3)
N9A—C26A	1.454 (2)	C25B—C26B	1.380 (3)
C21A—C26A	1.450 (2)	C25B—H25B	0.9300
C21A—C22A	1.454 (3)	O1WB—H1WB	0.8669
C22A—C23A	1.377 (3)	O1WB—H2WB	0.8651
C23A—C24A	1.395 (3)	C21—O1X	1.3577 (10)
C23A—H23A	0.9300	C21—O2X	1.3612 (10)
C24A—C25A	1.383 (3)	C21—H21A	0.9600
C25A—C26A	1.378 (2)	C21—H21B	0.9600
C25A—H25A	0.9300	C21—H21C	0.9600
O1WA—H1WA	0.8508	C21—H21D	0.9601
O1WA—H2WA	0.8529	C21—H21E	0.9601
Gd1B—O13B	2.2920 (13)	C21—H21F	0.9599
Gd1B—O6B	2.3771 (13)	O1X—H1XA	0.8200
Gd1B—O1WB	2.3844 (13)	O2X—H1XB	0.8200
			
N2A···O22B^i^	2.948 (3)	O9B···O25A^ii^	2.924 (2)
N2B···O25A^ii^	2.916 (2)	O17B···O24B^iii^	3.035 (3)
			
O13A—Gd1A—O6A	146.72 (4)	O6B—Gd1B—O7B	68.43 (4)
O13A—Gd1A—O1WA	75.58 (5)	O1WB—Gd1B—O7B	72.66 (5)
O6A—Gd1A—O1WA	132.29 (4)	O3B—Gd1B—O7B	134.81 (5)
O13A—Gd1A—O3A	74.46 (5)	O13B—Gd1B—O1B	81.08 (5)
O6A—Gd1A—O3A	74.17 (4)	O6B—Gd1B—O1B	127.90 (4)
O1WA—Gd1A—O3A	148.75 (5)	O1WB—Gd1B—O1B	68.98 (4)
O13A—Gd1A—O4A	103.27 (5)	O3B—Gd1B—O1B	101.48 (5)
O6A—Gd1A—O4A	72.79 (4)	O7B—Gd1B—O1B	76.26 (5)
O1WA—Gd1A—O4A	131.76 (5)	O13B—Gd1B—O2B	117.26 (5)
O3A—Gd1A—O4A	64.88 (4)	O6B—Gd1B—O2B	71.23 (5)
O13A—Gd1A—O7A	144.09 (5)	O1WB—Gd1B—O2B	127.23 (5)
O6A—Gd1A—O7A	68.48 (4)	O3B—Gd1B—O2B	64.45 (5)
O1WA—Gd1A—O7A	78.21 (5)	O7B—Gd1B—O2B	75.21 (5)
O3A—Gd1A—O7A	132.67 (4)	O1B—Gd1B—O2B	63.39 (4)
O4A—Gd1A—O7A	76.87 (4)	O13B—Gd1B—O5B	102.85 (5)
O13A—Gd1A—O5A	76.82 (5)	O6B—Gd1B—O5B	74.30 (4)
O6A—Gd1A—O5A	125.43 (5)	O1WB—Gd1B—O5B	64.47 (5)
O1WA—Gd1A—O5A	69.90 (4)	O3B—Gd1B—O5B	128.12 (5)
O3A—Gd1A—O5A	111.15 (4)	O7B—Gd1B—O5B	73.34 (5)
O4A—Gd1A—O5A	63.22 (4)	O1B—Gd1B—O5B	129.92 (4)
O7A—Gd1A—O5A	71.34 (4)	O2B—Gd1B—O5B	139.80 (5)
O13A—Gd1A—O1A	115.13 (5)	O13B—Gd1B—O4B	75.10 (5)
O6A—Gd1A—O1A	73.13 (4)	O6B—Gd1B—O4B	72.90 (4)
O1WA—Gd1A—O1A	65.41 (5)	O1WB—Gd1B—O4B	110.12 (5)
O3A—Gd1A—O1A	121.11 (5)	O3B—Gd1B—O4B	66.24 (4)
O4A—Gd1A—O1A	141.43 (4)	O7B—Gd1B—O4B	127.97 (4)
O7A—Gd1A—O1A	74.25 (5)	O1B—Gd1B—O4B	155.24 (4)
O5A—Gd1A—O1A	127.74 (4)	O2B—Gd1B—O4B	122.65 (4)
O13A—Gd1A—O2A	77.31 (5)	O5B—Gd1B—O4B	63.52 (4)
O6A—Gd1A—O2A	78.86 (5)	C1B—O1B—Gd1B	122.80 (11)
O1WA—Gd1A—O2A	101.63 (5)	C1B—O1B—H1OB	119.5
O3A—Gd1A—O2A	63.10 (4)	Gd1B—O1B—H1OB	116.1
O4A—Gd1A—O2A	125.57 (4)	C3B—O2B—C2B	112.64 (15)
O7A—Gd1A—O2A	132.38 (4)	C3B—O2B—Gd1B	119.06 (12)
O5A—Gd1A—O2A	154.05 (4)	C2B—O2B—Gd1B	116.83 (10)
O1A—Gd1A—O2A	63.43 (4)	C4B—O3B—C5B	117.09 (15)
C2A—O1A—Gd1A	117.66 (10)	C4B—O3B—Gd1B	117.22 (11)
C2A—O1A—H1OA	121.2	C5B—O3B—Gd1B	115.53 (10)
Gd1A—O1A—H1OA	121.2	C7B—O4B—C6B	112.49 (14)
C1A—O2A—C3A	111.27 (14)	C7B—O4B—Gd1B	120.06 (10)
C1A—O2A—Gd1A	121.82 (11)	C6B—O4B—Gd1B	116.47 (11)
C3A—O2A—Gd1A	120.70 (11)	C8B—O5B—Gd1B	118.23 (10)
C4A—O3A—C8A	116.74 (13)	C8B—O5B—H5OB	106.7
C4A—O3A—Gd1A	116.00 (10)	Gd1B—O5B—H5OB	121.0
C8A—O3A—Gd1A	121.43 (10)	C9B—O6B—Gd1B	139.38 (11)
C6A—O4A—C7A	112.60 (13)	N1B—O7B—Gd1B	138.21 (11)
C6A—O4A—Gd1A	121.51 (10)	C15B—O13B—Gd1B	141.15 (12)
C7A—O4A—Gd1A	114.86 (10)	O8B—N1B—O7B	121.40 (16)
C5A—O5A—Gd1A	119.68 (11)	O8B—N1B—C10B	119.14 (15)
C5A—O5A—H5OA	114.7	O7B—N1B—C10B	119.46 (15)
Gd1A—O5A—H5OA	125.5	O10B—N2B—O9B	123.20 (16)
C9A—O6A—Gd1A	137.01 (11)	O10B—N2B—C12B	118.89 (16)
N1A—O7A—Gd1A	139.42 (11)	O9B—N2B—C12B	117.88 (16)
C15A—O13A—Gd1A	138.11 (11)	O11B—N3B—O12B	124.03 (16)
O8A—N1A—O7A	121.72 (16)	O11B—N3B—C14B	117.75 (16)
O8A—N1A—C10A	119.21 (15)	O12B—N3B—C14B	118.20 (15)
O7A—N1A—C10A	119.04 (15)	O14B—N4B—O15B	124.07 (19)
O10A—N2A—O9A	122.84 (17)	O14B—N4B—C16B	118.19 (16)
O10A—N2A—C12A	118.80 (18)	O15B—N4B—C16B	117.74 (18)
O9A—N2A—C12A	118.30 (18)	O17B—N5B—O16B	124.09 (19)
O11A—N3A—O12A	123.95 (17)	O17B—N5B—C18B	118.13 (17)
O11A—N3A—C14A	117.41 (16)	O16B—N5B—C18B	117.78 (18)
O12A—N3A—C14A	118.63 (16)	O19B—N6B—O18B	124.67 (16)
O15A—N4A—O14A	124.46 (16)	O19B—N6B—C20B	118.20 (16)
O15A—N4A—C16A	117.52 (15)	O18B—N6B—C20B	117.13 (16)
O14A—N4A—C16A	118.01 (16)	O1B—C1B—C2B	106.55 (16)
O16A—N5A—O17A	123.64 (18)	O1B—C1B—H1BA	110.4
O16A—N5A—C18A	118.34 (17)	C2B—C1B—H1BA	110.4
O17A—N5A—C18A	118.00 (18)	O1B—C1B—H1BB	110.4
O19A—N6A—O18A	123.54 (18)	C2B—C1B—H1BB	110.4
O19A—N6A—C20A	118.57 (16)	H1BA—C1B—H1BB	108.6
O18A—N6A—C20A	117.88 (17)	O2B—C2B—C1B	108.43 (16)
O2A—C1A—C2A	106.13 (15)	O2B—C2B—H2BA	110.0
O2A—C1A—H1AA	110.5	C1B—C2B—H2BA	110.0
C2A—C1A—H1AA	110.5	O2B—C2B—H2BB	110.0
O2A—C1A—H1AB	110.5	C1B—C2B—H2BB	110.0
C2A—C1A—H1AB	110.5	H2BA—C2B—H2BB	108.4
H1AA—C1A—H1AB	108.7	O2B—C3B—C4B	108.57 (16)
O1A—C2A—C1A	108.34 (15)	O2B—C3B—H3BA	110.0
O1A—C2A—H2AA	110.0	C4B—C3B—H3BA	110.0
C1A—C2A—H2AA	110.0	O2B—C3B—H3BB	110.0
O1A—C2A—H2AB	110.0	C4B—C3B—H3BB	110.0
C1A—C2A—H2AB	110.0	H3BA—C3B—H3BB	108.4
H2AA—C2A—H2AB	108.4	O3B—C4B—C3B	107.09 (17)
O2A—C3A—C4A	107.84 (15)	O3B—C4B—H4BA	110.3
O2A—C3A—H3AA	110.1	C3B—C4B—H4BA	110.3
C4A—C3A—H3AA	110.1	O3B—C4B—H4BB	110.3
O2A—C3A—H3AB	110.1	C3B—C4B—H4BB	110.3
C4A—C3A—H3AB	110.1	H4BA—C4B—H4BB	108.6
H3AA—C3A—H3AB	108.5	O3B—C5B—C6B	109.37 (14)
O3A—C4A—C3A	105.85 (14)	O3B—C5B—H5BA	109.8
O3A—C4A—H4AA	110.6	C6B—C5B—H5BA	109.8
C3A—C4A—H4AA	110.6	O3B—C5B—H5BB	109.8
O3A—C4A—H4AB	110.6	C6B—C5B—H5BB	109.8
C3A—C4A—H4AB	110.6	H5BA—C5B—H5BB	108.2
H4AA—C4A—H4AB	108.7	O4B—C6B—C5B	106.48 (15)
O5A—C5A—C6A	107.10 (15)	O4B—C6B—H6BA	110.4
O5A—C5A—H5AA	110.3	C5B—C6B—H6BA	110.4
C6A—C5A—H5AA	110.3	O4B—C6B—H6BB	110.4
O5A—C5A—H5AB	110.3	C5B—C6B—H6BB	110.4
C6A—C5A—H5AB	110.3	H6BA—C6B—H6BB	108.6
H5AA—C5A—H5AB	108.6	O4B—C7B—C8B	106.22 (15)
O4A—C6A—C5A	106.64 (14)	O4B—C7B—H7BA	110.5
O4A—C6A—H6AA	110.4	C8B—C7B—H7BA	110.5
C5A—C6A—H6AA	110.4	O4B—C7B—H7BB	110.5
O4A—C6A—H6AB	110.4	C8B—C7B—H7BB	110.5
C5A—C6A—H6AB	110.4	H7BA—C7B—H7BB	108.7
H6AA—C6A—H6AB	108.6	O5B—C8B—C7B	108.86 (14)
O4A—C7A—C8A	108.83 (15)	O5B—C8B—H8BA	109.9
O4A—C7A—H7AA	109.9	C7B—C8B—H8BA	109.9
C8A—C7A—H7AA	109.9	O5B—C8B—H8BB	109.9
O4A—C7A—H7AB	109.9	C7B—C8B—H8BB	109.9
C8A—C7A—H7AB	109.9	H8BA—C8B—H8BB	108.3
H7AA—C7A—H7AB	108.3	O6B—C9B—C10B	125.98 (16)
O3A—C8A—C7A	104.41 (13)	O6B—C9B—C14B	122.12 (15)
O3A—C8A—H8AA	110.9	C10B—C9B—C14B	111.82 (14)
C7A—C8A—H8AA	110.9	C11B—C10B—C9B	124.59 (16)
O3A—C8A—H8AB	110.9	C11B—C10B—N1B	115.40 (15)
C7A—C8A—H8AB	110.9	C9B—C10B—N1B	119.99 (15)
H8AA—C8A—H8AB	108.9	C12B—C11B—C10B	118.05 (16)
O6A—C9A—C10A	126.04 (16)	C12B—C11B—H11B	121.0
O6A—C9A—C14A	121.60 (15)	C10B—C11B—H11B	121.0
C10A—C9A—C14A	112.27 (14)	C11B—C12B—C13B	121.77 (16)
C11A—C10A—C9A	124.20 (16)	C11B—C12B—N2B	119.60 (16)
C11A—C10A—N1A	115.87 (15)	C13B—C12B—N2B	118.63 (17)
C9A—C10A—N1A	119.93 (14)	C14B—C13B—C12B	117.98 (17)
C12A—C11A—C10A	117.97 (16)	C14B—C13B—H13B	121.0
C12A—C11A—H11A	121.0	C12B—C13B—H13B	121.0
C10A—C11A—H11A	121.0	C13B—C14B—C9B	124.87 (16)
C11A—C12A—C13A	122.05 (16)	C13B—C14B—N3B	116.05 (16)
C11A—C12A—N2A	119.35 (17)	C9B—C14B—N3B	119.08 (14)
C13A—C12A—N2A	118.60 (17)	O13B—C15B—C20B	124.78 (16)
C14A—C13A—C12A	117.88 (17)	O13B—C15B—C16B	122.86 (16)
C14A—C13A—H13A	121.1	C20B—C15B—C16B	112.22 (16)
C12A—C13A—H13A	121.1	C17B—C16B—C15B	124.94 (17)
C13A—C14A—C9A	124.99 (16)	C17B—C16B—N4B	117.34 (16)
C13A—C14A—N3A	117.08 (16)	C15B—C16B—N4B	117.70 (16)
C9A—C14A—N3A	117.93 (14)	C16B—C17B—C18B	118.02 (16)
O13A—C15A—C16A	123.02 (16)	C16B—C17B—H17B	121.0
O13A—C15A—C20A	124.34 (16)	C18B—C17B—H17B	121.0
C16A—C15A—C20A	112.48 (15)	C19B—C18B—C17B	121.77 (17)
C17A—C16A—C15A	125.07 (15)	C19B—C18B—N5B	119.65 (17)
C17A—C16A—N4A	116.28 (15)	C17B—C18B—N5B	118.57 (17)
C15A—C16A—N4A	118.43 (15)	C20B—C19B—C18B	118.34 (17)
C16A—C17A—C18A	117.67 (16)	C20B—C19B—H19B	120.8
C16A—C17A—H17A	121.2	C18B—C19B—H19B	120.8
C18A—C17A—H17A	121.2	C19B—C20B—C15B	124.58 (16)
C19A—C18A—C17A	121.76 (17)	C19B—C20B—N6B	116.97 (16)
C19A—C18A—N5A	119.02 (16)	C15B—C20B—N6B	118.27 (15)
C17A—C18A—N5A	119.18 (17)	O22B—N7B—O21B	122.95 (19)
C20A—C19A—C18A	118.81 (16)	O22B—N7B—C22B	117.86 (18)
C20A—C19A—H19A	120.6	O21B—N7B—C22B	119.16 (17)
C18A—C19A—H19A	120.6	O24B—N8B—O23B	124.03 (18)
C19A—C20A—C15A	123.98 (17)	O24B—N8B—C24B	118.31 (17)
C19A—C20A—N6A	116.84 (16)	O23B—N8B—C24B	117.65 (18)
C15A—C20A—N6A	119.05 (16)	O26B—N9B—O25B	123.34 (17)
O21A—N7A—O22A	123.80 (16)	O26B—N9B—C26B	118.20 (17)
O21A—N7A—C22A	118.47 (15)	O25B—N9B—C26B	118.39 (17)
O22A—N7A—C22A	117.66 (16)	O20B—C21B—C26B	124.48 (15)
O23A—N8A—O24A	123.88 (17)	O20B—C21B—C22B	123.17 (16)
O23A—N8A—C24A	118.09 (16)	C26B—C21B—C22B	112.28 (15)
O24A—N8A—C24A	118.01 (17)	C23B—C22B—C21B	124.42 (17)
O26A—N9A—O25A	122.42 (16)	C23B—C22B—N7B	116.63 (15)
O26A—N9A—C26A	119.82 (14)	C21B—C22B—N7B	118.94 (16)
O25A—N9A—C26A	117.76 (15)	C22B—C23B—C24B	118.60 (16)
O20A—C21A—C26A	124.27 (16)	C22B—C23B—H23B	120.7
O20A—C21A—C22A	124.02 (15)	C24B—C23B—H23B	120.7
C26A—C21A—C22A	111.71 (15)	C23B—C24B—C25B	121.72 (17)
C23A—C22A—C21A	124.54 (16)	C23B—C24B—N8B	119.13 (17)
C23A—C22A—N7A	115.74 (16)	C25B—C24B—N8B	119.05 (18)
C21A—C22A—N7A	119.69 (15)	C26B—C25B—C24B	118.74 (18)
C22A—C23A—C24A	118.65 (17)	C26B—C25B—H25B	120.6
C22A—C23A—H23A	120.7	C24B—C25B—H25B	120.6
C24A—C23A—H23A	120.7	C25B—C26B—C21B	123.78 (16)
C25A—C24A—C23A	121.38 (17)	C25B—C26B—N9B	116.07 (16)
C25A—C24A—N8A	119.25 (16)	C21B—C26B—N9B	120.13 (15)
C23A—C24A—N8A	119.23 (16)	Gd1B—O1WB—H1WB	124.5
C26A—C25A—C24A	119.15 (16)	Gd1B—O1WB—H2WB	115.3
C26A—C25A—H25A	120.4	H1WB—O1WB—H2WB	102.7
C24A—C25A—H25A	120.4	O1X—C21—H21A	108.4
C25A—C26A—C21A	124.43 (16)	O1X—C21—H21B	109.9
C25A—C26A—N9A	114.98 (15)	O1X—C21—H21C	110.2
C21A—C26A—N9A	120.54 (15)	H21A—C21—H21B	109.5
Gd1A—O1WA—H1WA	109.3	H21A—C21—H21C	109.5
Gd1A—O1WA—H2WA	109.6	H21B—C21—H21C	109.5
H1WA—O1WA—H2WA	107.4	O2X—C21—H21D	106.8
O13B—Gd1B—O6B	145.32 (5)	O2X—C21—H21E	110.5
O13B—Gd1B—O1WB	74.54 (5)	O2X—C21—H21F	111.1
O6B—Gd1B—O1WB	129.49 (4)	H21D—C21—H21E	109.5
O13B—Gd1B—O3B	75.49 (5)	H21D—C21—H21F	109.5
O6B—Gd1B—O3B	79.67 (5)	H21E—C21—H21F	109.5
O1WB—Gd1B—O3B	149.62 (5)	C21—O1X—H1XA	109.5
O13B—Gd1B—O7B	145.06 (5)	C21—O2X—H1XB	109.5
			
O13A—Gd1A—O1A—C2A	37.40 (14)	O13B—Gd1B—O1B—C1B	−122.69 (14)
O6A—Gd1A—O1A—C2A	−107.95 (13)	O6B—Gd1B—O1B—C1B	36.19 (15)
O1WA—Gd1A—O1A—C2A	96.27 (13)	O1WB—Gd1B—O1B—C1B	160.53 (15)
O3A—Gd1A—O1A—C2A	−49.01 (14)	O3B—Gd1B—O1B—C1B	−49.58 (15)
O4A—Gd1A—O1A—C2A	−136.79 (12)	O7B—Gd1B—O1B—C1B	84.10 (14)
O7A—Gd1A—O1A—C2A	−179.73 (13)	O2B—Gd1B—O1B—C1B	3.98 (13)
O5A—Gd1A—O1A—C2A	129.96 (12)	O5B—Gd1B—O1B—C1B	137.98 (13)
O2A—Gd1A—O1A—C2A	−22.20 (12)	O4B—Gd1B—O1B—C1B	−106.79 (16)
O13A—Gd1A—O2A—C1A	−135.34 (14)	O13B—Gd1B—O2B—C3B	−52.70 (15)
O6A—Gd1A—O2A—C1A	68.06 (13)	O6B—Gd1B—O2B—C3B	90.62 (15)
O1WA—Gd1A—O2A—C1A	−63.22 (14)	O1WB—Gd1B—O2B—C3B	−143.56 (14)
O3A—Gd1A—O2A—C1A	145.82 (14)	O3B—Gd1B—O2B—C3B	3.34 (14)
O4A—Gd1A—O2A—C1A	127.29 (13)	O7B—Gd1B—O2B—C3B	162.44 (15)
O7A—Gd1A—O2A—C1A	21.35 (15)	O1B—Gd1B—O2B—C3B	−115.76 (15)
O5A—Gd1A—O2A—C1A	−130.95 (13)	O5B—Gd1B—O2B—C3B	122.97 (14)
O1A—Gd1A—O2A—C1A	−8.52 (13)	O4B—Gd1B—O2B—C3B	36.53 (16)
O13A—Gd1A—O2A—C3A	74.76 (13)	O13B—Gd1B—O2B—C2B	88.06 (13)
O6A—Gd1A—O2A—C3A	−81.84 (13)	O6B—Gd1B—O2B—C2B	−128.62 (13)
O1WA—Gd1A—O2A—C3A	146.88 (13)	O1WB—Gd1B—O2B—C2B	−2.79 (15)
O3A—Gd1A—O2A—C3A	−4.08 (13)	O3B—Gd1B—O2B—C2B	144.10 (14)
O4A—Gd1A—O2A—C3A	−22.61 (15)	O7B—Gd1B—O2B—C2B	−56.79 (13)
O7A—Gd1A—O2A—C3A	−128.55 (13)	O1B—Gd1B—O2B—C2B	25.01 (12)
O5A—Gd1A—O2A—C3A	79.15 (16)	O5B—Gd1B—O2B—C2B	−96.27 (14)
O1A—Gd1A—O2A—C3A	−158.42 (14)	O4B—Gd1B—O2B—C2B	177.30 (12)
O13A—Gd1A—O3A—C4A	−49.70 (12)	O13B—Gd1B—O3B—C4B	156.89 (15)
O6A—Gd1A—O3A—C4A	119.05 (13)	O6B—Gd1B—O3B—C4B	−47.52 (14)
O1WA—Gd1A—O3A—C4A	−32.70 (17)	O1WB—Gd1B—O3B—C4B	147.19 (14)
O4A—Gd1A—O3A—C4A	−162.88 (13)	O7B—Gd1B—O3B—C4B	−2.59 (17)
O7A—Gd1A—O3A—C4A	157.79 (11)	O1B—Gd1B—O3B—C4B	79.37 (14)
O5A—Gd1A—O3A—C4A	−118.52 (12)	O2B—Gd1B—O3B—C4B	26.50 (14)
O1A—Gd1A—O3A—C4A	60.61 (13)	O5B—Gd1B—O3B—C4B	−108.01 (14)
O2A—Gd1A—O3A—C4A	33.71 (11)	O4B—Gd1B—O3B—C4B	−123.26 (15)
O13A—Gd1A—O3A—C8A	102.51 (13)	O13B—Gd1B—O3B—C5B	−58.70 (12)
O6A—Gd1A—O3A—C8A	−88.75 (13)	O6B—Gd1B—O3B—C5B	96.89 (13)
O1WA—Gd1A—O3A—C8A	119.51 (13)	O1WB—Gd1B—O3B—C5B	−68.40 (17)
O4A—Gd1A—O3A—C8A	−10.67 (12)	O7B—Gd1B—O3B—C5B	141.82 (12)
O7A—Gd1A—O3A—C8A	−50.01 (15)	O1B—Gd1B—O3B—C5B	−136.23 (12)
O5A—Gd1A—O3A—C8A	33.68 (14)	O2B—Gd1B—O3B—C5B	170.90 (14)
O1A—Gd1A—O3A—C8A	−147.19 (12)	O5B—Gd1B—O3B—C5B	36.40 (14)
O2A—Gd1A—O3A—C8A	−174.08 (14)	O4B—Gd1B—O3B—C5B	21.14 (12)
O13A—Gd1A—O4A—C6A	54.64 (13)	O13B—Gd1B—O4B—C7B	−127.71 (13)
O6A—Gd1A—O4A—C6A	−159.66 (13)	O6B—Gd1B—O4B—C7B	65.83 (13)
O1WA—Gd1A—O4A—C6A	−27.76 (15)	O1WB—Gd1B—O4B—C7B	−60.74 (13)
O3A—Gd1A—O4A—C6A	120.14 (14)	O3B—Gd1B—O4B—C7B	151.85 (14)
O7A—Gd1A—O4A—C6A	−88.45 (13)	O7B—Gd1B—O4B—C7B	22.56 (14)
O5A—Gd1A—O4A—C6A	−12.94 (12)	O1B—Gd1B—O4B—C7B	−143.97 (13)
O1A—Gd1A—O4A—C6A	−130.76 (12)	O2B—Gd1B—O4B—C7B	119.18 (13)
O2A—Gd1A—O4A—C6A	138.39 (12)	O5B—Gd1B—O4B—C7B	−14.79 (12)
O13A—Gd1A—O4A—C7A	−86.70 (12)	O13B—Gd1B—O4B—C6B	90.85 (13)
O6A—Gd1A—O4A—C7A	59.00 (11)	O6B—Gd1B—O4B—C6B	−75.60 (12)
O1WA—Gd1A—O4A—C7A	−169.10 (11)	O1WB—Gd1B—O4B—C6B	157.82 (12)
O3A—Gd1A—O4A—C7A	−21.20 (11)	O3B—Gd1B—O4B—C6B	10.41 (12)
O7A—Gd1A—O4A—C7A	130.21 (12)	O7B—Gd1B—O4B—C6B	−118.88 (12)
O5A—Gd1A—O4A—C7A	−154.28 (13)	O1B—Gd1B—O4B—C6B	74.59 (16)
O1A—Gd1A—O4A—C7A	87.90 (13)	O2B—Gd1B—O4B—C6B	−22.25 (14)
O2A—Gd1A—O4A—C7A	−2.95 (13)	O5B—Gd1B—O4B—C6B	−156.22 (14)
O13A—Gd1A—O5A—C5A	−130.62 (12)	O13B—Gd1B—O5B—C8B	49.29 (13)
O6A—Gd1A—O5A—C5A	21.89 (13)	O6B—Gd1B—O5B—C8B	−95.01 (12)
O1WA—Gd1A—O5A—C5A	150.14 (13)	O1WB—Gd1B—O5B—C8B	114.97 (13)
O3A—Gd1A—O5A—C5A	−63.30 (13)	O3B—Gd1B—O5B—C8B	−32.22 (14)
O4A—Gd1A—O5A—C5A	−18.14 (11)	O7B—Gd1B—O5B—C8B	−166.67 (13)
O7A—Gd1A—O5A—C5A	66.23 (12)	O1B—Gd1B—O5B—C8B	138.34 (12)
O1A—Gd1A—O5A—C5A	117.65 (12)	O2B—Gd1B—O5B—C8B	−126.76 (12)
O2A—Gd1A—O5A—C5A	−135.02 (12)	O4B—Gd1B—O5B—C8B	−16.62 (11)
O13A—Gd1A—O6A—C9A	−166.15 (15)	O13B—Gd1B—O6B—C9B	−159.64 (15)
O1WA—Gd1A—O6A—C9A	−25.58 (19)	O1WB—Gd1B—O6B—C9B	−33.91 (19)
O3A—Gd1A—O6A—C9A	173.81 (18)	O3B—Gd1B—O6B—C9B	155.67 (18)
O4A—Gd1A—O6A—C9A	105.78 (17)	O7B—Gd1B—O6B—C9B	8.27 (16)
O7A—Gd1A—O6A—C9A	23.45 (16)	O1B—Gd1B—O6B—C9B	59.10 (18)
O5A—Gd1A—O6A—C9A	68.83 (18)	O2B—Gd1B—O6B—C9B	89.32 (17)
O1A—Gd1A—O6A—C9A	−55.87 (17)	O5B—Gd1B—O6B—C9B	−69.65 (17)
O2A—Gd1A—O6A—C9A	−121.24 (17)	O4B—Gd1B—O6B—C9B	−136.20 (18)
O13A—Gd1A—O7A—N1A	−164.30 (15)	O13B—Gd1B—O7B—N1B	−172.77 (15)
O6A—Gd1A—O7A—N1A	6.72 (17)	O6B—Gd1B—O7B—N1B	19.24 (17)
O1WA—Gd1A—O7A—N1A	151.92 (18)	O1WB—Gd1B—O7B—N1B	166.36 (18)
O3A—Gd1A—O7A—N1A	−33.6 (2)	O3B—Gd1B—O7B—N1B	−29.1 (2)
O4A—Gd1A—O7A—N1A	−69.72 (17)	O1B—Gd1B—O7B—N1B	−121.73 (18)
O5A—Gd1A—O7A—N1A	−135.54 (18)	O2B—Gd1B—O7B—N1B	−56.08 (17)
O1A—Gd1A—O7A—N1A	84.43 (18)	O5B—Gd1B—O7B—N1B	98.56 (18)
O2A—Gd1A—O7A—N1A	56.86 (19)	O4B—Gd1B—O7B—N1B	64.03 (19)
O6A—Gd1A—O13A—C15A	167.51 (15)	O6B—Gd1B—O13B—C15B	167.67 (16)
O1WA—Gd1A—O13A—C15A	16.53 (17)	O1WB—Gd1B—O13B—C15B	28.21 (18)
O3A—Gd1A—O13A—C15A	−172.48 (19)	O3B—Gd1B—O13B—C15B	−146.72 (19)
O4A—Gd1A—O13A—C15A	−113.71 (18)	O7B—Gd1B—O13B—C15B	7.5 (2)
O7A—Gd1A—O13A—C15A	−27.8 (2)	O1B—Gd1B—O13B—C15B	−42.32 (18)
O5A—Gd1A—O13A—C15A	−55.76 (18)	O2B—Gd1B—O13B—C15B	−96.10 (18)
O1A—Gd1A—O13A—C15A	70.01 (19)	O5B—Gd1B—O13B—C15B	86.77 (19)
O2A—Gd1A—O13A—C15A	122.27 (18)	O4B—Gd1B—O13B—C15B	144.50 (19)
Gd1A—O7A—N1A—O8A	152.05 (15)	Gd1B—O7B—N1B—O8B	142.15 (15)
Gd1A—O7A—N1A—C10A	−29.8 (2)	Gd1B—O7B—N1B—C10B	−38.9 (2)
C3A—O2A—C1A—C2A	−173.25 (16)	Gd1B—O1B—C1B—C2B	−29.5 (2)
Gd1A—O2A—C1A—C2A	34.3 (2)	C3B—O2B—C2B—C1B	93.15 (19)
Gd1A—O1A—C2A—C1A	48.18 (19)	Gd1B—O2B—C2B—C1B	−50.05 (18)
O2A—C1A—C2A—O1A	−50.3 (2)	O1B—C1B—C2B—O2B	48.4 (2)
C1A—O2A—C3A—C4A	−175.54 (16)	C2B—O2B—C3B—C4B	−172.34 (18)
Gd1A—O2A—C3A—C4A	−22.8 (2)	Gd1B—O2B—C3B—C4B	−30.0 (2)
C8A—O3A—C4A—C3A	148.50 (16)	C5B—O3B—C4B—C3B	164.41 (17)
Gd1A—O3A—C4A—C3A	−57.95 (16)	Gd1B—O3B—C4B—C3B	−51.7 (2)
O2A—C3A—C4A—O3A	49.0 (2)	O2B—C3B—C4B—O3B	50.3 (2)
Gd1A—O5A—C5A—C6A	43.90 (19)	C4B—O3B—C5B—C6B	94.69 (19)
C7A—O4A—C6A—C5A	−178.99 (15)	Gd1B—O3B—C5B—C6B	−49.76 (18)
Gd1A—O4A—C6A—C5A	38.88 (19)	C7B—O4B—C6B—C5B	178.08 (16)
O5A—C5A—C6A—O4A	−49.7 (2)	Gd1B—O4B—C6B—C5B	−37.65 (18)
C6A—O4A—C7A—C8A	−95.23 (18)	O3B—C5B—C6B—O4B	55.6 (2)
Gd1A—O4A—C7A—C8A	49.54 (17)	C6B—O4B—C7B—C8B	−176.32 (16)
C4A—O3A—C8A—C7A	−170.05 (15)	Gd1B—O4B—C7B—C8B	40.84 (19)
Gd1A—O3A—C8A—C7A	37.94 (18)	Gd1B—O5B—C8B—C7B	43.71 (18)
O4A—C7A—C8A—O3A	−53.99 (19)	O4B—C7B—C8B—O5B	−52.1 (2)
Gd1A—O6A—C9A—C10A	−27.5 (3)	Gd1B—O6B—C9B—C10B	−12.3 (3)
Gd1A—O6A—C9A—C14A	156.26 (13)	Gd1B—O6B—C9B—C14B	171.18 (12)
O6A—C9A—C10A—C11A	176.71 (16)	O6B—C9B—C10B—C11B	174.38 (16)
C14A—C9A—C10A—C11A	−6.7 (2)	C14B—C9B—C10B—C11B	−8.8 (2)
O6A—C9A—C10A—N1A	−4.6 (3)	O6B—C9B—C10B—N1B	−7.3 (3)
C14A—C9A—C10A—N1A	171.90 (15)	C14B—C9B—C10B—N1B	169.49 (14)
O8A—N1A—C10A—C11A	26.8 (2)	O8B—N1B—C10B—C11B	27.3 (2)
O7A—N1A—C10A—C11A	−151.38 (15)	O7B—N1B—C10B—C11B	−151.68 (15)
O8A—N1A—C10A—C9A	−151.94 (16)	O8B—N1B—C10B—C9B	−151.20 (16)
O7A—N1A—C10A—C9A	29.9 (2)	O7B—N1B—C10B—C9B	29.8 (2)
C9A—C10A—C11A—C12A	0.6 (3)	C9B—C10B—C11B—C12B	3.6 (3)
N1A—C10A—C11A—C12A	−178.12 (15)	N1B—C10B—C11B—C12B	−174.84 (15)
C10A—C11A—C12A—C13A	6.1 (3)	C10B—C11B—C12B—C13B	5.8 (3)
C10A—C11A—C12A—N2A	−174.37 (15)	C10B—C11B—C12B—N2B	−174.67 (15)
O10A—N2A—C12A—C11A	−179.86 (18)	O10B—N2B—C12B—C11B	−167.39 (17)
O9A—N2A—C12A—C11A	2.8 (3)	O9B—N2B—C12B—C11B	14.5 (2)
O10A—N2A—C12A—C13A	−0.4 (3)	O10B—N2B—C12B—C13B	12.1 (3)
O9A—N2A—C12A—C13A	−177.72 (17)	O9B—N2B—C12B—C13B	−165.94 (16)
C11A—C12A—C13A—C14A	−5.7 (3)	C11B—C12B—C13B—C14B	−8.7 (3)
N2A—C12A—C13A—C14A	174.76 (16)	N2B—C12B—C13B—C14B	171.80 (16)
C12A—C13A—C14A—C9A	−1.5 (3)	C12B—C13B—C14B—C9B	2.5 (3)
C12A—C13A—C14A—N3A	179.02 (16)	C12B—C13B—C14B—N3B	−177.93 (15)
O6A—C9A—C14A—C13A	−176.05 (17)	O6B—C9B—C14B—C13B	−177.35 (16)
C10A—C9A—C14A—C13A	7.2 (2)	C10B—C9B—C14B—C13B	5.7 (2)
O6A—C9A—C14A—N3A	3.5 (2)	O6B—C9B—C14B—N3B	3.0 (2)
C10A—C9A—C14A—N3A	−173.24 (15)	C10B—C9B—C14B—N3B	−173.88 (15)
O11A—N3A—C14A—C13A	−56.5 (2)	O11B—N3B—C14B—C13B	−48.4 (2)
O12A—N3A—C14A—C13A	122.63 (19)	O12B—N3B—C14B—C13B	129.93 (18)
O11A—N3A—C14A—C9A	124.0 (2)	O11B—N3B—C14B—C9B	131.3 (2)
O12A—N3A—C14A—C9A	−56.9 (2)	O12B—N3B—C14B—C9B	−50.4 (2)
Gd1A—O13A—C15A—C16A	94.2 (2)	Gd1B—O13B—C15B—C20B	83.0 (2)
Gd1A—O13A—C15A—C20A	−80.9 (2)	Gd1B—O13B—C15B—C16B	−92.4 (2)
O13A—C15A—C16A—C17A	−170.43 (16)	O13B—C15B—C16B—C17B	171.62 (18)
C20A—C15A—C16A—C17A	5.2 (2)	C20B—C15B—C16B—C17B	−4.2 (3)
O13A—C15A—C16A—N4A	4.1 (2)	O13B—C15B—C16B—N4B	−6.9 (3)
C20A—C15A—C16A—N4A	179.66 (14)	C20B—C15B—C16B—N4B	177.29 (15)
O15A—N4A—C16A—C17A	42.2 (2)	O14B—N4B—C16B—C17B	131.0 (2)
O14A—N4A—C16A—C17A	−137.38 (18)	O15B—N4B—C16B—C17B	−49.0 (3)
O15A—N4A—C16A—C15A	−132.75 (17)	O14B—N4B—C16B—C15B	−50.5 (2)
O14A—N4A—C16A—C15A	47.6 (2)	O15B—N4B—C16B—C15B	129.6 (2)
C15A—C16A—C17A—C18A	−2.5 (3)	C15B—C16B—C17B—C18B	4.1 (3)
N4A—C16A—C17A—C18A	−177.11 (15)	N4B—C16B—C17B—C18B	−177.42 (17)
C16A—C17A—C18A—C19A	−0.3 (3)	C16B—C17B—C18B—C19B	−2.0 (3)
C16A—C17A—C18A—N5A	177.40 (17)	C16B—C17B—C18B—N5B	179.54 (17)
O16A—N5A—C18A—C19A	−173.9 (2)	O17B—N5B—C18B—C19B	10.8 (3)
O17A—N5A—C18A—C19A	7.7 (3)	O16B—N5B—C18B—C19B	−169.86 (19)
O16A—N5A—C18A—C17A	8.3 (3)	O17B—N5B—C18B—C17B	−170.7 (2)
O17A—N5A—C18A—C17A	−170.1 (2)	O16B—N5B—C18B—C17B	8.7 (3)
C17A—C18A—C19A—C20A	0.0 (3)	C17B—C18B—C19B—C20B	0.4 (3)
N5A—C18A—C19A—C20A	−177.77 (17)	N5B—C18B—C19B—C20B	178.90 (17)
C18A—C19A—C20A—C15A	3.3 (3)	C18B—C19B—C20B—C15B	−0.8 (3)
C18A—C19A—C20A—N6A	179.03 (17)	C18B—C19B—C20B—N6B	−175.80 (16)
O13A—C15A—C20A—C19A	170.02 (17)	O13B—C15B—C20B—C19B	−173.23 (17)
C16A—C15A—C20A—C19A	−5.5 (2)	C16B—C15B—C20B—C19B	2.5 (3)
O13A—C15A—C20A—N6A	−5.7 (3)	O13B—C15B—C20B—N6B	1.7 (3)
C16A—C15A—C20A—N6A	178.81 (15)	C16B—C15B—C20B—N6B	177.44 (15)
O19A—N6A—C20A—C19A	145.80 (18)	O19B—N6B—C20B—C19B	−143.92 (18)
O18A—N6A—C20A—C19A	−33.7 (3)	O18B—N6B—C20B—C19B	36.4 (2)
O19A—N6A—C20A—C15A	−38.2 (2)	O19B—N6B—C20B—C15B	40.8 (2)
O18A—N6A—C20A—C15A	142.31 (18)	O18B—N6B—C20B—C15B	−138.88 (17)
O20A—C21A—C22A—C23A	176.06 (17)	O20B—C21B—C22B—C23B	−169.20 (18)
C26A—C21A—C22A—C23A	−4.2 (2)	C26B—C21B—C22B—C23B	7.8 (3)
O20A—C21A—C22A—N7A	−6.0 (3)	O20B—C21B—C22B—N7B	10.3 (3)
C26A—C21A—C22A—N7A	173.65 (15)	C26B—C21B—C22B—N7B	−172.69 (16)
O21A—N7A—C22A—C23A	148.56 (17)	O22B—N7B—C22B—C23B	29.3 (3)
O22A—N7A—C22A—C23A	−28.6 (2)	O21B—N7B—C22B—C23B	−148.77 (18)
O21A—N7A—C22A—C21A	−29.5 (2)	O22B—N7B—C22B—C21B	−150.2 (2)
O22A—N7A—C22A—C21A	153.35 (16)	O21B—N7B—C22B—C21B	31.7 (3)
C21A—C22A—C23A—C24A	2.8 (3)	C21B—C22B—C23B—C24B	−4.2 (3)
N7A—C22A—C23A—C24A	−175.13 (16)	N7B—C22B—C23B—C24B	176.27 (17)
C22A—C23A—C24A—C25A	0.7 (3)	C22B—C23B—C24B—C25B	−1.1 (3)
C22A—C23A—C24A—N8A	176.37 (16)	C22B—C23B—C24B—N8B	−177.27 (17)
O23A—N8A—C24A—C25A	176.42 (18)	O24B—N8B—C24B—C23B	178.1 (2)
O24A—N8A—C24A—C25A	−1.9 (3)	O23B—N8B—C24B—C23B	−0.8 (3)
O23A—N8A—C24A—C23A	0.6 (3)	O24B—N8B—C24B—C25B	1.8 (3)
O24A—N8A—C24A—C23A	−177.70 (18)	O23B—N8B—C24B—C25B	−177.03 (19)
C23A—C24A—C25A—C26A	−2.3 (3)	C23B—C24B—C25B—C26B	2.0 (3)
N8A—C24A—C25A—C26A	−177.95 (16)	N8B—C24B—C25B—C26B	178.14 (17)
C24A—C25A—C26A—C21A	0.5 (3)	C24B—C25B—C26B—C21B	2.4 (3)
C24A—C25A—C26A—N9A	177.76 (16)	C24B—C25B—C26B—N9B	−175.79 (17)
O20A—C21A—C26A—C25A	−177.75 (17)	O20B—C21B—C26B—C25B	170.14 (18)
C22A—C21A—C26A—C25A	2.6 (2)	C22B—C21B—C26B—C25B	−6.9 (2)
O20A—C21A—C26A—N9A	5.1 (3)	O20B—C21B—C26B—N9B	−11.7 (3)
C22A—C21A—C26A—N9A	−174.60 (15)	C22B—C21B—C26B—N9B	171.29 (16)
O26A—N9A—C26A—C25A	155.17 (18)	O26B—N9B—C26B—C25B	158.42 (19)
O25A—N9A—C26A—C25A	−23.9 (2)	O25B—N9B—C26B—C25B	−18.7 (3)
O26A—N9A—C26A—C21A	−27.4 (3)	O26B—N9B—C26B—C21B	−19.9 (3)
O25A—N9A—C26A—C21A	153.52 (17)	O25B—N9B—C26B—C21B	163.01 (18)

Symmetry codes: (i) *x*+1, *y*, *z*; (ii) *x*+1, *y*, *z*+1; (iii) −*x*+1, −*y*, −*z*+1.

### Hydrogen-bond geometry (Å, °)

**Table:**

*D*—H···*A*	*D*—H	H···*A*	*D*···*A*	*D*—H···*A*
O1A—H1OA···O9A^iv^	0.93	2.18	2.822 (2)	125
O1A—H1OA···O10A^iv^	0.93	2.23	3.156 (2)	173
O1A—H1OA···N2A^iv^	0.93	2.51	3.374 (2)	154
O5A—H5OA···O20A^i^	0.85	1.92	2.706 (2)	153
O5A—H5OA···O21A^i^	0.85	2.58	3.132 (2)	123
O1WA—H1WA···O26A^i^	0.85	2.54	2.879 (2)	105
O1WA—H1WA···N6A	0.85	2.56	3.344 (2)	154
O1WA—H2WA···O20A^i^	0.85	1.84	2.686 (2)	173
O1B—H1OB···O20B^i^	0.84	1.88	2.661 (2)	155
O5B—H5OB···O9B^v^	0.87	1.95	2.805 (2)	168
O5B—H5OB···N2B^v^	0.87	2.62	3.451 (2)	160
O1WB—H1WB···O21B^i^	0.87	2.39	2.727 (2)	104
O1WB—H2WB···O20B^i^	0.86	1.74	2.592 (2)	170
O1WB—H2WB···O21B^i^	0.86	2.42	2.727 (2)	101
O1X—H1XA···O10B^vi^	0.82	2.52	3.194 (4)	140
O1X—H1XA···O26A^i^	0.82	2.26	2.811 (4)	125
C4A—H4AB···O13A	0.97	2.42	2.992 (2)	117
C11B—H11B···O11B^i^	0.93	2.51	3.429 (2)	170
C5A—H5AB···O21A^i^	0.97	2.37	3.016 (2)	123
C7A—H7AA···O6A	0.97	2.56	3.072 (2)	113
C7A—H7AA···O23B	0.97	2.49	3.004 (2)	113
C7A—H7AB···O18B^iii^	0.97	2.39	3.278 (2)	152
C17A—H17A···O23A^vii^	0.93	2.32	3.235 (3)	167
C19B—H19B···O24B^iii^	0.93	2.39	3.255 (3)	155
C21—H21B···O10B^vi^	0.96	2.50	3.236 (3)	133
C1B—H1BA···O26B^i^	0.97	2.43	3.085 (3)	125
C3B—H3BB···O15A^iii^	0.97	2.59	3.111 (3)	114
C5B—H5BB···O20B	0.97	2.60	3.222 (2)	122
C6B—H6BB···O12B	0.97	2.51	3.375 (3)	149
C7B—H7BA···O12B	0.97	2.56	3.407 (3)	146

Symmetry codes: (iv) −*x*+2, −*y*+1, −*z*+1; (i) *x*+1, *y*, *z*; (v) −*x*+2, −*y*+1, −*z*+2; (vi) *x*, *y*, *z*−1; (iii) −*x*+1, −*y*, −*z*+1; (vii) −*x*+1, −*y*, −*z*.
